# FoxP1 is a transcriptional repressor associated with cancer cachexia that induces skeletal muscle wasting and weakness

**DOI:** 10.1002/jcsm.12666

**Published:** 2021-02-01

**Authors:** Daria Neyroud, Rachel L. Nosacka, Chandler S. Callaway, Jose G. Trevino, Hui Hu, Sarah M. Judge, Andrew R. Judge

**Affiliations:** ^1^ Department of Physical Therapy University of Florida Gainesville FL USA; ^2^ Department of Surgery University of Florida Gainesville FL USA; ^3^ Department of Microbiology University of Alabama at Birmingham Birmingham AL USA

**Keywords:** Muscle atrophy, Cancer cachexia, Muscle regeneration, Muscle weakness, Pancreatic cancer

## Abstract

**Background:**

Skeletal muscle wasting is a devastating consequence of cancer that affects up to 80% of cancer patients and associates with reduced survival. Herein, we investigated the biological significance of Forkhead box P1 (FoxP1), a transcriptional repressor that we demonstrate is up‐regulated in skeletal muscle in multiple models of cancer cachexia and in cachectic cancer patients.

**Methods:**

Inducible, skeletal muscle‐specific FoxP1 over‐expressing (FoxP1^iSkmTg/Tg^) mice were generated through crossing conditional *Foxp1a* transgenic mice with HSA‐MCM mice that express tamoxifen‐inducible Cre recombinase under control of the skeletal muscle actin promoter. To determine the requirement of FoxP1 for cancer‐induced skeletal muscle wasting, FoxP1‐shRNA was packaged and targeted to muscles using AAV9 delivery prior to inoculation of mice with Colon‐26 Adenocarcinoma (C26) cells.

**Results:**

Up‐regulation of FoxP1 in adult skeletal muscle was sufficient to induce features of cachexia, including 15% reduction in body mass (*P* < 0.05), and a 16–27% reduction in skeletal muscle mass (*P* < 0.05) that was characterized by a 20% reduction in muscle fibre cross‐sectional area of type IIX/B muscle fibres (*P* = 0.020). Skeletal muscles from FoxP1^iSkmTg/Tg^ mice also showed significant damage and myopathy characterized by the presence of centrally nucleated myofibres, extracellular matrix expansion, and were 19–26% weaker than controls (*P* < 0.05). Transcriptomic analysis revealed FoxP1 as a potent transcriptional repressor of skeletal muscle gene expression, with enrichment of pathways related to skeletal muscle structure and function, growth signalling, and cell quality control. Because FoxP1 functions, at least in part, as a transcriptional repressor through its interaction with histone deacetylase proteins, we treated FoxP1^iSkmTg/Tg^ mice with Trichostatin A, and found that this completely prevented the loss of muscle mass (*p* = 0.007) and fibre atrophy (*P* < 0.001) in the *tibialis anterior*. In the context of cancer, FoxP1 knockdown blocked the cancer‐induced repression of myocyte enhancer factor 2 (MEF2)‐target genes critical to muscle differentiation and repair, improved muscle ultrastructure, and attenuated muscle fibre atrophy by 50% (*P* < 0.05).

**Conclusions:**

In summary, we identify FoxP1 as a novel repressor of skeletal muscle gene expression that is increased in cancer cachexia, whose up‐regulation is sufficient to induce skeletal muscle wasting and weakness, and required for the normal wasting response to cancer.

## Introduction

Cancer‐induced cachexia is a metabolic condition defined by the involuntary reduction in body weight resulting from the loss of muscle mass, with or without concomitant loss of fat.^1^ This devastating condition affects up to 80% of all cancer patients^2^ and is causative in the functional impairments and reduced quality of life experienced by these patients (refer to Fearon *et al*.^1^ for review). Furthermore, the presence of cachexia limits cancer treatment options due to increased treatment toxicity^2^, ^3^, ^4^ and is associated with reduced survival.^5^ Indeed, it is suggested that cachexia itself accounts for approximately 20–30% of all cancer‐related deaths.^1^, ^5^, ^6^ Unfortunately, there are currently no effective therapeutic treatments for cachexia, which is in part due to our incomplete understanding of cancer‐induced cachexia pathophysiology.

The current understanding of cancer cachexia indicates that factors secreted by tumours together with factors secreted as a result of the tumour–host interaction initiate chronic systemic inflammation and metabolic disturbances, which in turn trigger muscle wasting (refer to Baracos *et al*.^7^ for a detailed review of the current understanding of the pathophysiology of cancer cachexia). Using experimental models of cancer cachexia, several intracellular signalling proteins, including the atrophy‐associated Forkhead box O (FoxO) transcription factors, were shown to mediate this muscle wasting pathology.^8^, ^9^, ^10^, ^11^, ^12^, ^13^, ^14^, ^15^ Skeletal muscle expression of FoxO is increased in multiple models of cancer cachexia, including the subcutaneous Lewis Lung Carcinoma (LLC) and Colon 26 Adenocarcinoma (C26) models, the Yoshida ascites hepatoma model, and in multiple orthotopic models of pancreatic cancer cachexia, including the patient‐derived xenograft model, in which a fragment of a patient tumour is directly sutured to the mouse pancreas.^12^, ^13^, ^16^, ^17^, ^18^ These findings are relevant to patients, because FoxO1 is also up‐regulated in muscle biopsies from cancer patients who exhibit cachexia.^19^, ^20^ Subsequent work from our laboratory further established that FoxO transcriptional activity is necessary for cancer‐induced muscle atrophy,^15^ thus pinpointing FoxO‐regulated genes as key mediators of the wasting phenotype.^13^, ^15^ Using genome‐wide transcriptomic analyses of muscle from tumour‐bearing mice in which FoxO activity was either intact or blocked via transduction with a dominant negative FoxO construct, we identified FoxO as a key upstream activator of multiple transcription factors belonging to the basic Leucine Zipper transcription factor family, which was the most highly enriched functional annotation.^15^ Included among the basic Leucine Zipper transcription factors up‐regulated by FoxO was Forkhead box P1 (FoxP1), which is a negative regulator of gene transcription. Indeed, through its interaction with and recruitment of transcriptional repressor complexes to gene promoters, FoxP1 exerts potent transcriptional repression in several cell types.^21^, ^22^, ^23^ This is potentially of interest, because recently published RNA‐seq data in the skeletal muscle of cachectic cancer patients revealed gene repression as the predominant direction of change of differentially expressed genes.^24^

Although the role of FoxP1 in skeletal muscle remains unknown, FoxP1 has been shown to be involved in cell proliferation and differentiation in a variety of other tissues,^25^, ^26^, ^27^, ^28^, ^29^, ^30^ including myocytes, in which FoxP1 deletion results in embryonic heart failure and death.^31^ In addition, mutations in FoxP1 gene have been documented in patients presenting with congenital heart defects,^32^ speech delay, contractures, and hypertonia.^33^ Alterations in FoxP1 expression have also been associated with poor cancer‐survival prognosis,^34^, ^35^, ^36^ atherosclerosis,^37^ Huntington disease,^38^ heart failure,^39^ and cardiomyocyte hypertrophy.^40^ Based on our current knowledge of the role of FoxP1, coupled with its concomitant increase in the skeletal muscle of mice bearing atrophy‐inducing tumours, we hypothesized that FoxP1 is a key mediator of cancer‐induced skeletal muscle wasting. To test this hypothesis, we generated conditional skeletal muscle‐specific FoxP1 over‐expressing mice to determine the sufficiency of FoxP1 to induce muscle wasting and weakness, and through FoxP1 knockdown, further determined the requirement of FoxP1 for the muscle atrophy phenotype induced by tumour burden.

## Materials and methods

### Patient biopsies

Biopsies from the *rectus abdominis* muscle used for protein analyses were obtained from non‐cancer control patients undergoing abdominal surgery and from cachectic patients diagnosed with pancreatic ductal adenocarcinoma (PDAC) undergoing tumour resection surgery. All procedures were compliant with an approved Institutional Review Board protocol, and written informed consent was obtained from all patients. PDAC patients presenting a >5% body mass loss in combination with muscle depletion (i.e. low skeletal muscle index and SMI^41^) were classified as cachectic. Patient characteristics, including SMI and skeletal muscle microarray data for *FOXP1* mRNA, were extracted from our previous work (Series GSE130563 ^20^).

### Animals

All animal procedures were approved by the University of Florida Institutional Animal Care and Use Committee. Animals were provided with *ad libitum* access to food (either a standard diet or a tamoxifen diet, see below) and water, and housed in a temperature‐controlled and humidity‐controlled facility on a 12 h of light/dark cycle.

#### Models of cancer cachexia

CD2F1 male mice were purchased from Charles River laboratory (Wilmington, Massachusetts, USA) and injected subcutaneously in each flank with 5 × 10^5^ C26 tumour cells (cultured as described in Roberts et al^42^) diluted in 100 μL of sterile phosphate‐buffered saline (PBS) (C26), or injected with sterile PBS alone (vehicle). Male C57BL/6J mice were purchased from Jackson laboratory (Bar Harbor, Maine, USA) and subcutaneously injected in each flank with 2.5 × 10^6^ LLC cells diluted in 100 μL of sterile PBS (LLC) or equivalent volume of sterile PBS (vehicle). LLC cells were cultured in Dulbecco's Modified Eagle Medium (DMEM) supplemented with 10% foetal bovine serum, 1% penicillin, 1% streptomycin at 37°C in a 5% CO_2_ humidified chamber. Male immunocompromised NOD.Cg‐Prkdc^scid^ IL2rg^tm1Wjl^/SzJ (NSG) mice were purchased from Jackson laboratory and inoculated either subcutaneously (flank) or orthotopically (ortho) with L3.6 pancreas‐liver (L3.6pl) cells as described previously.^16^ All muscles were harvested when mice reached Institutional Animal Care and Use Committees‐mandated humane endpoint based on tumour size and body condition scores.

#### Plasmid injections

Male C57BL/6J mice purchased from Jackson laboratory or male Sprague Dawley Rats purchased from Charles River (200 g) were used for experiments involving plasmid electroporation.

#### Inducible over‐expression of FoxP1 in skeletal muscles

Mice with conditional transgenic expression of FoxP1 were created as previously described.^25^ Briefly, a FoxP1A transgene preceded by an in‐frame stop sequence flanked by two loxP sites was knocked‐in at the *Rosa*26 locus.^25^ These mice were subsequently bred with HSA‐MCM mice (purchased from Jackson laboratory) in which the expression of a chimeric Cre recombinase containing a mutated oestrogen receptor ligand‐binding domain is driven by the alpha‐skeletal actin promoter,^43^ to generate CRE+ tamoxifen‐inducible skeletal muscle‐specific FoxP1 over‐expressing (FoxP1^iSkmTg/Tg^) mice. CRE‐ littermate controls treated with tamoxifen (tamoxifen controls) or FoxP1^iSkmTg/Tg^ mice treated with vehicle (genetic controls) were used as controls, where indicated.

### Tamoxifen treatment

FoxP1^iSkmTg/Tg^ and CRE‐ littermate controls were injected intraperitoneally with 80 mg/kg of tamoxifen (ThermoFisher, Waltham, Massachusetts, USA) diluted in 100 μL of corn oil on five consecutive days, whereas genetic controls were injected with 100 μL of corn oil (vehicle). On the last day of tamoxifen injection, standard diet was substituted with a diet supplemented with 250 mg of tamoxifen per kilogram of diet (TD.130856, Envigo, Huntingdon, UK). Mice were then maintained on this diet until tissue harvest, for up to 6 weeks, depending on the experiment.

### Trichostatin treatment

Mice were intraperitoneally injected daily with 0.6 mg of trichostatin (TSA) per kilogram of body mass (MilliporeSigma) diluted in 100 μL of sterile PBS. For plasmid injection studies, TSA treatments began on the day of plasmid injection. For studies using FoxP1^iSkmTg/Tg^ mice, TSA was delivered throughout the duration of the tamoxifen treatment, that is during the 23 days immediately preceding tissue harvest.

### Adeno‐associated virus (AAV) and plasmid injections

AAV2/9‐mCherry‐U6‐mFOXO1‐shRNA, AAV2/9‐mCherry‐U6‐mFOXP1‐shRNA and AAV9‐GFP‐U6‐scrmb‐shRNA were purchased from Vector Biolabs (Malvern, Pennsylvania, USA). All vectors were diluted in 20 μL of Lactated Ringer solution to a final concentration of 1 × 10^11^ vector genome per muscle and injected through the skin into the mid‐belly of *tibialis anterior* 2 weeks before C26 tumour cell inoculation, as described previously.^15^

The FoxP1 plasmids were obtained from (i) Dr D'Mello (University of Texas, Dallas, USA) and has been described in Louis Sam Titus *et al*.^38^ and (ii) Addgene (pcDNA3.1 Foxp1A‐#16362, Addgene Watertown, Massachusetts). The pEGFP‐C1 plasmid (i.e. empty vector and EV) was purchased from Clontech laboratories (#6084‐1, Mountain View, California, USA). The plasmid coding for the constitutively active FoxO1 (1099 pcDNA GFP FKHR AAA, #9023) and the 3XMEF2‐luc reporter plasmid (plasmid # 32967) were obtained from Addgene (Watertown, Massachusetts, USA). The FoxP1 promoter reporter plasmid (MPRM17622‐PG02) was purchased from Genecopoeia (Rockville, Maryland, USA). Plasmid DNA, amplified using bacterial cultures, was isolated using an Endotoxin‐free MaxiPrep kit (Qiagen, Hilden, Germany). Plasmid DNA was subsequently precipitated with ethanol and diluted in sterile PBS for *in vivo* injections and electroporation as previously detailed,^44^ to a concentration of 1 μg/μL, except for pEGFP‐C1 that was diluted to 0.2 μg/μL. Each *tibialis anterior* was injected with 20 μL.

### Cardiotoxin injury

*Tibialis anterior* muscles of CRE+ FoxP1^iSkmTg/Tg^ mice and CRE‐ littermate controls were directly injected with 10 μM of cardiotoxin (MilliporeSigma, Burlington, Massachusetts, USA) diluted in 1xPBS (75 μL for male mice and 50 μL for female mice), which induces widespread degeneration and *de novo* muscle regeneration. On the same day as cardiotoxin injections, treatments with tamoxifen were initiated, and injured muscles harvested 24 days later.

### *Ex vivo* muscle function assessment

*Ex vivo* muscle function was assessed at the Physiological Assessment Core of the University of Florida on freshly isolated diaphragm strips and *soleus* muscles (as previously described^45^, ^46^) from ≥9‐week‐old FoxP1^iSkmTg/Tg^ mice and CRE‐ littermate controls, 3.5 weeks following initiation of tamoxifen treatment. Briefly, muscles were mounted on a force transducer placed in a 22°C bath of Ringers solution that was gas‐equilibrated with 95% O_2_–5% CO_2_ attached to a dual mode force transducer (Aurora Scientific, Ontario, Canada). After determination of muscle optimal length, maximum isometric twitch and tetanic forces were acquired using a single supramaximal stimulation and a 500‐ms stimulation train at 150 Hz, respectively, with a 5 min of rest period between each set. Subsequently, diaphragm strips were stimulated at 5, 10, 30, 50, 70, 90, 110, 130, and 150 Hz to build a force–frequency relationship, whereas *solei* were stimulated for once per second for 10 min (200‐μs pulse width, 100 Hz, 330‐ms duration) in order to determine resistance to fatigue.

### C2C12 culture

Murine C2C12 were obtained from the American Type Culture Collection (Manassas, Virginia, USA). C2C12 were cultured as previously described.^47^ Briefly, C2C12 were incubated at 37°C in the presence of 5% CO_2_ in DMEM supplemented with 10% foetal bovine serum and 1% penicillin/streptomycin. Myoblasts were differentiated in DMEM supplemented with 2% horse serum (Thermofisher).

### RNA isolation, RT‐qPCR, and microarray analyses

RNA isolation was performed by homogenization of muscle samples or C2C12 cells in Trizol (ThermoFisher) followed by extraction with chloroform and precipitation with isopropanol. One microgram of RNA was retro‐transcribed using the SuperScript IV Reverse Transcriptase kit (Thermofisher) according to the manufacturer guidelines. Complementary DNA was used for amplification of the following target genes using the TaqMan probe‐based chemistry (Thermofisher): FoxP1 (Mm00474848_m1), Mef2c (Mm01340842_m1), Ky (Mm00600373_m1), Myom2 (Mm00500665_m1), Lmod3 (Mm01182486_m1), Casq1 (Mm00486725_m1), Casq2 (Mm00486742_m1), Ryr1 (Mm01175211_m1), Atp1b2 (Mm00442612_m1), Myod1 (Mm00440387_m1), Jph2 (Mm00517621_m1), and Acta1 (Mm01253177_m1). Gene expression quantification was performed using a relative standard curve method. Microarray analysis was performed by Boston University School of Medicine Microarray Core Facility using the Mouse Gene 2.0ST array. Briefly, for determination of FoxP1 target genes, RNA samples from *tibialis anterior* muscles (*n* = 3 mice per group) transfected with FoxP1A or GFP plasmids were respectively pooled and sent to Boston University Microarray Core for amplification, labelling, and hybridization on the Mouse Gene 2.0ST array. Similarly, to assess the extent to which FoxP1 knockdown prevents C26‐induced transcriptional dysregulation, RNA extracted from *tibialis anterior* muscles of C26 tumour‐bearing and non‐tumour bearing mice transfected with mFOXP1‐shRNA or scrambled‐shRNA were pooled (*n* = 3 per condition) and sent to Boston University Microarray Core. Mouse Gene 2.0ST CEL files were normalized to produce gene‐level expression values using the implementation of the Robust Multiarray Average ^48^ in the *affy* package (Version 1.36.1)^49^ included in the Bioconductor software suite (Version 2.12)^50^ and an Entrez gene‐specific probeset mapping (17.0.0) from the Molecular and Behavioural Neuroscience Institute (Brainarray) at the University of Michigan.^51^ Array quality was assessed by computing Relative Log Expression using the affyPLM package (Version 1.34.0),^52^ or by performing Robust Multiarray Average normalization using Expression Console (build 1.4.1.46) and computing area under the curve values. Principal component analysis was performed using the prcomp R function with expression values that had been normalized across all samples to a mean of zero and a standard deviation of one. Human homologues of mouse genes were identified using HomoloGene (Version 68).^53^ All microarray analyses were performed using the R environment for statistical computing (Version 2.15.1). For follow‐up enrichment bioinformatics analyses, genes were sorted based on their fold change (most down‐regulated to most up‐regulated gene) compared with the empty vector group and the full list of genes was entered into String (Version 11^54^). Additional bioinformatics analyses were conducted to identify enriched ‘Canonical Pathways’ and ‘Diseases and Functions’ through the Ingenuity Pathway Analysis software (QIAGEN Inc., https://www.qiagenbioinformatics.com/products/ingenuity-pathway-analysis,^55^) using a *P* value cut‐off of 0.001 and a *z*‐score ≤−2 or ≥2. In addition, genes down‐regulated two‐fold or greater in response to C26, which were increased by two‐fold or more in the presence of FoxP1‐shRNA, were further analysed with DAVID (Version 6.8) to identify the most highly enriched non‐redundant functional annotations. Genes were also analysed using Gene Set Enrichment Analysis, using the C3 (regulatory target gene sets) collection within the Molecular Signatures Database (MSigDB 7.0) to identify enriched transcription factor binding motifs among gene promoters.^56^, ^57^ CEL files and expression values were deposited into MIAME compliant NCBI Gene Expression Omnibus with accession number #GSE153068 (Link: https://www.ncbi.nlm.nih.gov/geo/query/acc.cgi?acc=GSE153068 Token: etadawiaxzknbcj).

### Immunohistochemistry

Skeletal muscle tissues were embedded in optimal cutting temperature compound and frozen in liquid isopentane cooled in liquid nitrogen before being stored at −80°C. Prior to cryosectioning, samples were equilibrated at −20°C, and then, 10‐μm skeletal muscle sections were cut with a microtome cryostat. Sections were stained with haematoxylin/eosin (H&E) as described previously^58^ or stored at −80°C. Fibres presenting internalized nuclei on H&E images were considered to be regenerating and counted using ImageJ software. Alizarin Red S stain was used to identify calcium deposits in skeletal muscle fibres. Briefly, muscle sections were thawed and air‐dried for 30 min at room temperature, hydrated in 100% and 70% ethanol, stained in Alizarin red solution for 30 s, dehydrated in acetone and acetone : xylene (1:1), and cleared in xylene. Periodic acid‐Schiff was performed according to manufacturer recommendation (Newcomer Supply, Middleton, Wisconsin, USA). Immunofluorescence imaging was used to examine fibre typology. Briefly, sections were thawed and air‐dried for 30 min, rehydrated for 5 min in PBS, blocked for 30 min in 25% SuperBlock blocking buffer diluted in PBS (ThermoFisher), incubated for 1 h at room temperature with antibodies against myosin heavy chain I (BA‐D5, DSHB) and myosin heavy chain IIA (SC‐71), washed 3 × 5 min with PBS, incubated 1 h at room temperature with appropriate secondary antibodies and wheat germ agglutinin (WGA) conjugated with Alexa Fluor 594 (Invitrogen, Carlsbad, California, USA), and washed 3 × 5 min with PBS. To assess myofibre damage and morphology, air‐dried sections were rehydrated 5 min in PBS, blocked for 30 min in 25% SuperBlock blocking buffer diluted in PBS (ThermoFisher), incubated for 1 h at room temperature with WGA conjugated with Alexa Fluor 594 and Alexa Fluor 488‐conjugated goat anti‐mouse immunoglobulin G (IgG) antibody (Invitrogen, Carlsbad, California, USA) and washed 3 × 5 min with PBS. Sections were then imaged with a Leica DM5000B or Leica TCS SP8 microscope (Leica Microsystems, Bannockburn IL). ImageJ was used for all image analyses. Skeletal muscle fibre cross‐sectional area was measured by a semi‐automated threshold analysis using the WGA signal to delineate the borders of individual fibres. A total of 600–1400 (mean: ~1000), 230–775 (mean: ~500), and 1200–2600 (mean: ~2000) fibres were traced for every diaphragm, *soleus*, and *tibialis anterior* section, respectively. The WGA signal was also used to detect increased extracellular matrix/fibrosis, by quantifying the percentage of muscle area occupied by WGA‐positive staining.^59^ Skeletal muscle fibre damage was assessed through the identification of fibres presenting intracellular WGA and/or IgG staining.^59^, ^60^

### Transmission electron microscopy

Muscles for transmission electron microscopy (TEM) were sliced, fixed, and processed by the University of Florida Interdisciplinary Center for Biotechnology Research Electron Microscopy core. Briefly, samples were fixed in Trump's fixative (EMS, Hatfield, PA), processed with the aid of a Pelco BioWave Pro laboratory microwave (Ted Pella, Redding, CA, USA), and washed in 0.1 M of sodium cacodylate, pH 7.22, post fixed with buffered 2% OsO4, water washed and dehydrated in a graded ethanol series from 25% through 100% with 5% increments. Dehydrated samples were infiltrated in LRWhite Medium and Z6040 embedding primer (EMS, Hatfield, PA) in 50%, 100% and cured at 60°C for 48 h. Semi‐thick sections (500 nm) were stained with toluidine blue. Ultra‐thin sections were collected on carbon coated Formvar nickel slot grids (EMS, Hatfield, PA) post‐stained with 2% aqueous uranyl acetate and Reynold's lead citrate. Sections were examined with a FEI Tecnai G2 Spirit Twin TEM (FEI Corp., Hillsboro, OR), and digital images were acquired with a Gatan UltraScan 2 × 2 k camera and Digital Micrograph software (Gatan Inc., Pleasanton, CA).

### Western blotting

Snap frozen muscles were homogenized in ice‐cold RIPA buffer (MilliporeSigma) supplemented with protease inhibitors (Fisher Scientific, Hampton, NH, USA), phosphatase inhibitors (Fisher Scientific) and sodium butyrate, and centrifuged for 10 min at 10 000*g* at 4°C. Protein concentration was measured via a BCA assay (ThermoFisher). Samples were mixed in a 1:1 ratio with 2X Laemmli buffer supplemented with 5% of ß‐mercaptoethanol, denatured for 5 min at 90°C, and 24 μg of protein was loaded to a 4–15% TGX Stain‐free gel (Biorad, Hercules, CA, USA). Following protein electrophoresis, gels were activated by being exposed to UV light for 1 min. Thereafter, proteins were transferred to a nitrocellulose membrane using the Transblot turbo transfer system (Biorad). Membranes were subsequently blocked for 1 h in 5% bovine serum albumin diluted into TBS supplemented with 1% of Tween 20 (TBS‐T), incubated with FoxP1 (1:1000, ab32010, Abcam, Cambridge, UK) or GAPDH (1:2000, 14C10, Cell Signaling, Danvers, MA, USA) antibody for 1 h at room temperature, washed 3 × 5 min in TBS‐T, incubated 1 h in the dark with appropriate secondary antibodies (LI‐COR, Biosciences, Lincoln, NE, USA), and washed 3 × 5 min with TBS‐T before being imaged with an Odyssey Imaging System (LI‐COR, Biosciences). Signal intensity for FoxP1 was quantified using the Image Studio Lab software (LI‐COR, Biosciences) and normalized to GAPDH or total protein content.

### Statistical analyses

Depending on data normality, tested with Shapiro–Wilk test, the following parametric or non‐parametric statistical tests were used. Comparisons between two groups were conducted with two‐tailed *t*‐test or Mann–Whitney U test. In instances where more than two groups needed to be compared, one‐way analysis of variance (ANOVA) or Kruskal–Wallis one‐way ANOVA were performed. Two‐way mixed ANOVAs [groups (CRE‐ littermate controls vs. FoxP1^iSkmTg/Tg^) × treatment (vehicle vs. tamoxifen)] were used to test for differences in tissue masses as well as to evaluate differences between groups (CRE‐ littermate controls vs. FoxP1^iSkmTg/Tg^) in forces evoked at different frequencies or throughout the fatiguing protocol. When ANOVAs detected differences, Tukey (for one‐way ANOVA), Dunn's (for Kruskal–Wallis one‐way ANOVA), and Sidak (two‐way ANOVA) *post hoc* analyses were used to test for differences among pairs of means. The alpha level of significance was set to 0.05. Data are reported as mean ± standard error. All statistical analyses were performed with SigmaPlot software for Windows (version 11, Systat, Chicago, IL, USA) and Prism 8 for MacOS X (GraphPad Software, La Jolla, CA, USA). Prism 8 was also used for figures.

## Results

### FoxP1 is transcriptionally up‐regulated in multiple models of cancer cachexia, through a FoxO1‐dependent mechanism

**Figure 1:**
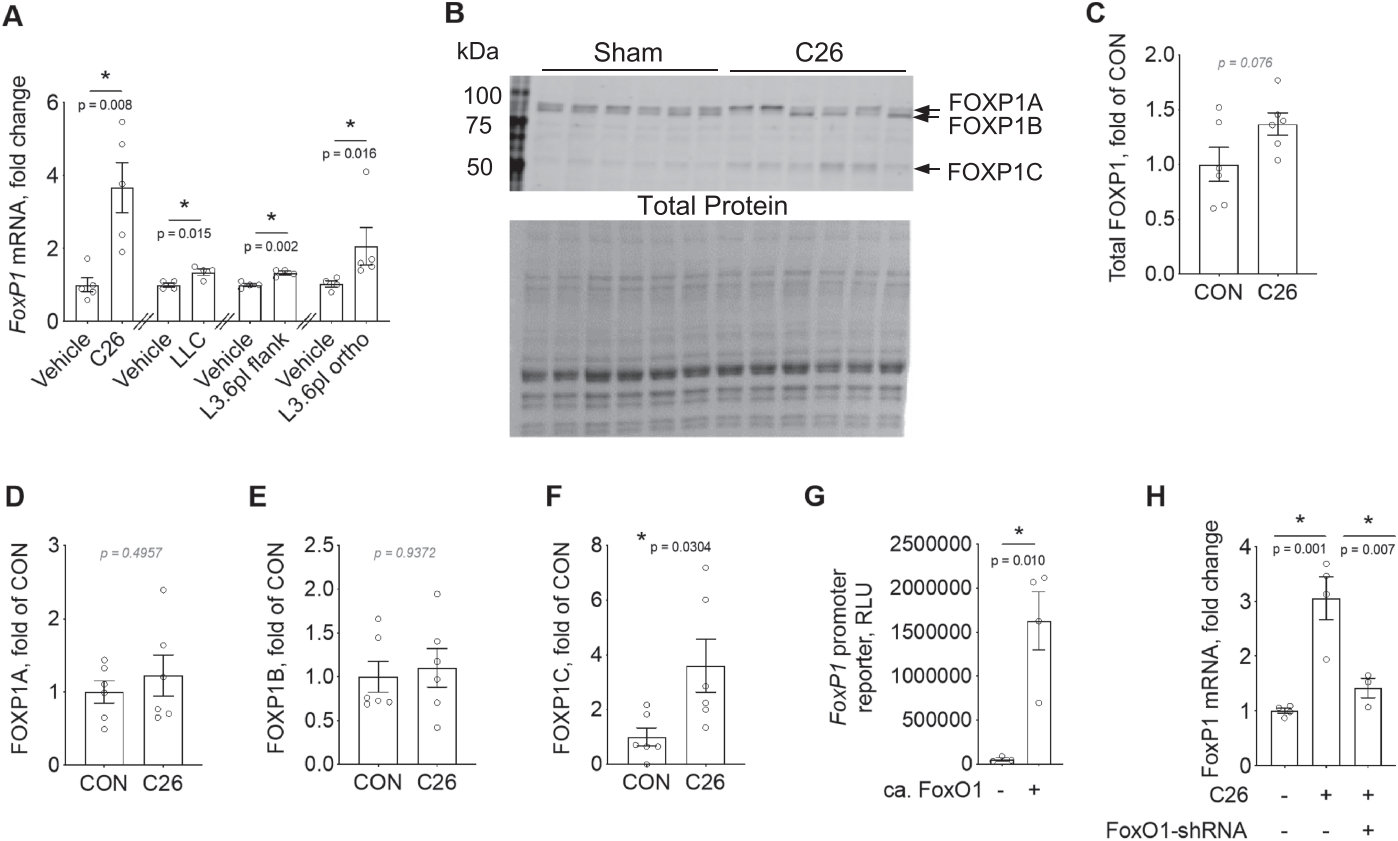
FoxP1 is up‐regulated in multiple models of cancer cachexia in a FoxO1‐dependent manner. (A) Changes in *FoxP1* mRNA in mouse skeletal muscle in various experimental models of cancer cachexia, including the subcutaneous murine colon 26 adenocarcinoma (C26) and Lewis lung carcinoma (LLC) models, and the human pancreas‐liver (L3.6pl) xenograft models, in which human L3.6pl cells were injected into the flank or orthotopically (ortho) into the pancreas. Note that independent unpaired two‐tailed *t*‐tests were performed for the different cachexia models. (B–F) FOXP1 protein expression in skeletal muscle of C26 tumour‐bearing and non‐tumour‐bearing (Sham) mice. In panel (C), total FOXP1 expression was quantified by summing the signal from FOXP1A, FOXP1B, and FOXP1C and normalizing to total protein. (G) Transfection of rat *solei* with a constitutively active FoxO1 expression plasmid (ca. FoxO1) is sufficient to increase FoxP1 transcription, *in vivo*, as highlighted by its ability to increase a luciferase reporter gene driven by the FoxP1 promoter (RLU = relative light unit). (H) FoxO1 knockdown in mouse *tibialis anterior*, through transduction of muscles with AAV9 vector encoding FoxO1‐shRNA (or scrambled‐shRNA), prevents the C26‐induced upregulation of *FoxP1* mRNA. For panels (A)–(G), depending on data distribution, unpaired two‐tailed *t*‐tests or Mann–Whitney tests were performed to test for statistical differences between groups. A one‐way analysis of variance (ANOVA) was conducted to test for statistical differences between means in panel (H). Data are reported as mean ± SEM.

Using reverse transcription–quantitative polymerase chain reaction (RT‐qPCR), we first confirmed that *FoxP1* is increased in skeletal muscle of cachectic mice bearing C26 tumours, and further established that *FoxP1* is elevated in several additional mouse models of cancer cachexia, including the subcutaneous LLC and L3.6pl models, as well as in the orthotopic L3.6pl model of pancreatic cancer cachexia (*Figure* 1A). We further determined through western blotting, the protein expression of FOXP1 in skeletal muscle of sham and C26 tumour‐bearing mice—which revealed the likely presence of multiple FOXP1 isoforms, including full length FOXP1 (~95 kDa), and at least two truncated versions that likely correspond to the smaller FOXP1 isoforms expressed in mice.^61^ Compared with sham, C26 mice showed notable variations in the abundance of the different FOXP1 isoforms present in muscle, with a numerical increase in total FOXP1 (*P* = 0.076) and a significant increase in the smallest FOXP1 isoform (*Figure* 1B–F). These data therefore align with our RT‐qPCR data, and together support the notion that FoxP1 is up‐regulated in muscle of cachectic tumour‐bearing mice.

We previously identified, through an unbiased microarray analysis, that *FoxP1* is a downstream target of FoxO, which is increased in the skeletal muscle of C26 tumour‐bearing mice.^15^ Of the FoxO family members, FoxO1 is the predominant isoform increased in skeletal muscle of both cachectic tumour‐bearing mice and cachectic cancer patients.^12^, ^13^, ^19^, ^20^ We therefore determined whether FoxO1 is sufficient to increase FoxP1 transcription and required for its up‐regulation in response to tumour burden. Rodent skeletal muscles were transfected with an empty vector or constitutively active FoxO1 expression plasmid plus a FoxP1 promoter reporter and harvested 4 days later. Compared with empty vector, FoxO1 significantly increased transcription from the FoxP1 promoter reporter (*Figure* 1G). In line with this finding, blocking the cancer‐induced up‐regulation of FoxO1 via AAV2/9 delivery of FoxO1‐shRNA prevented the C26‐induced increase in *FoxP1* mRNA (*Figure* 1H)—thus demonstrating the sufficiency and requirement of FoxO1 for FoxP1 up‐regulation.

### Skeletal muscle‐specific induction of FoxP1 induces body and skeletal muscle wasting

**Figure 2:**
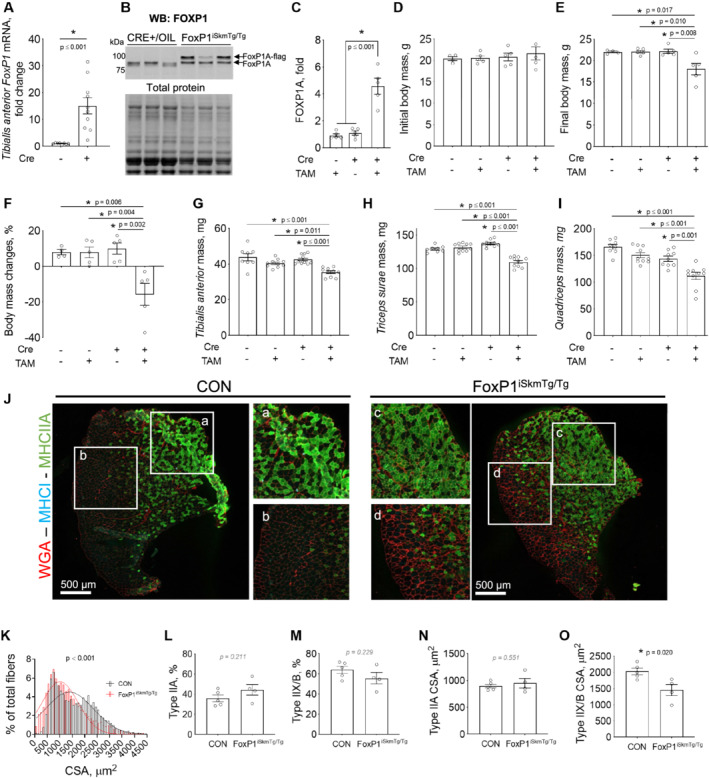
Inducible, skeletal muscle‐specific FoxP1 over‐expression induces body and skeletal muscle wasting. FoxP1 over‐expression was induced by injecting mice intraperitoneally with tamoxifen for five consecutive days, followed by maintenance on a tamoxifen diet until study endpoint. Mice were euthanized following 6 weeks of treatment, or upon reaching IACUC‐mandated endpoint, based on cachexia development. (A–C) Tamoxifen treatment (started at 9–15 weeks of age) increases FoxP1 mRNA (A) and protein (B–C) expression in CRE+ FoxP1^iSkmTg/Tg^ mice compared with controls. (D–I) Skeletal muscle‐specific FoxP1 over‐expression induces body wasting (D–F) and skeletal muscle wasting (G–I). TAM = tamoxifen. (J) Representative images of *tibialis anterior* cross‐sections stained for wheat germ agglutinin (WGA), myosin heavy chain type I (MHCI), and myosin heavy chain type IIA (MHCIIA). CON = genetic controls. (K) Quantification of *tibialis anterior* muscle fibre cross‐sectional area (CSA) shows a leftward shift in fibre sizes in response to FoxP1 over‐expression. CSA data were binned and fit with a Gaussian least squares regression. Significance was determined by calculating the extra sum‐of‐squares *F* test. (L–M) Quantification of type IIA (L) and type IIX/B (M) fibre percentage in genetic control (CON) and CRE+ FoxP1^iSkmTg/Tg^ *tibialis anterior*. (N–O) Quantification of type IIA (N) and type IIX/B (O) muscle fibre CSA showing type IIX/B fibre atrophy in *tibialis anterior* muscles over‐expressing FoxP1. Statistical differences were determined by conducting unpaired two‐tailed *t*‐tests or Mann–Whitney tests in panels (A) and (L–O), one‐way ANOVA in panel (C) and two‐way ANOVAs in panels (D)–(I). Data shown are from female mice and are reported as mean ± SEM. Note that data from male CRE+ FoxP1^iSkmTg/Tg^ mice and CRE‐ littermate controls are presented in *Figure* *S3*.

To determine the physiological significance of FoxP1 up‐regulation in skeletal muscle, we utilized plasmid injection and electroporation to transfect muscle with a FoxP1 expression plasmid (or an empty vector to serve as control), along with a GFP expression plasmid, to visualize transfected fibres. Skeletal muscle transfected with FoxP1 showed a 10% reduction in mass, and significantly reduced skeletal muscle fibre CSA 7 days post‐transfection, indicating that FoxP1 up‐regulation in skeletal muscle is sufficient to induce muscle fibre atrophy (*Figure* S1A–B). To substantiate and extend these findings, we generated mice in which FoxP1 over‐expression could be induced in skeletal muscles (FoxP1^iSkmTg/Tg^ mice) via treatment with tamoxifen (*Figure* 2A–C). A 15‐fold increase in *FoxP1* mRNA (*Figure* 2A) and a four‐fold increase in the protein expression of full length FOXP1 (*Figures* 2B–C and *S2*) were observed in response to tamoxifen treatment in skeletal muscles of FoxP1^iSkmTg/Tg^ mice. Compared with both CRE‐ littermate controls treated with tamoxifen, and to FoxP1^iSkmTg/Tg^ mice treated with vehicle, FoxP1^iSkmTg/Tg^ mice treated with tamoxifen showed body wasting reflected by an average loss of 15% of their original body mass (*Figure* 2D–F). Moreover, the body wasting in FoxP1^iSkmTg/Tg^ was associated with significant skeletal muscle wasting highlighted by lower muscle masses in the *tibialis anterior* (−16%), *gastrocnemius complex*/*triceps surae* (−17%), and quadriceps (−27%) of female (*Figure* 2G–I) and male FoxP1^iSkmTg/Tg^ mice (*Figure* S3). Noteworthy, FoxP1 over‐expression in skeletal muscle also caused fat wasting in several, but not all FoxP1^iSkmTg/Tg^ mice (*Figures* S3 *and* S4B), whereas heart and spleen masses were not affected (*Figure* S3E,G and S4A,C). Overall, these data show that sustained FoxP1 over‐expression in skeletal muscle is sufficient to induce body, muscle, and fat wasting, although the latter was variable.

To further determine whether the muscle wasting observed in FoxP1^iSkmTg/Tg^ mice is associated with muscle fibre atrophy, we measured *tibialis anterior* fibre cross‐sectional area (*Figure* 2J–O). Compared with genetic controls, FoxP1^iSkmTg/Tg^ mice showed a leftward shift in their fibre size distribution (*Figure* 2K); a similar shift was observed in FoxP1^iSkmTg/Tg^ diaphragm muscles (*Figure* *S5*). To determine whether this fibre size reduction was fibre‐type specific and associated with a shift in fibre type composition, we immunostained *tibialis anterior* cross‐sections to distinguish between myosin heavy chain I (type I), myosin heavy chain IIA (type IIA), and myosin heavy chain IIX/B (Type IIX/B) fibres. Type I fibres accounted for less than 1% of total fibres in FoxP1^iSkmTg/Tg^ mice and genetic controls and were therefore excluded from further analyses. Type IIA fibres represented approximately 43% of total fibres in FoxP1^iSkmTg/Tg^ vs. 35% in genetic controls (*Figure* 2L), whereas Type IIX/B fibres accounted for 57% of total fibres in FoxP1^iSkmTg/Tg^ vs. 62% in genetic controls (*Figure* 2M), neither of which were significantly different between strains. Fibre‐type specific analyses of muscle fibre size further demonstrated significant myofibre atrophy of type IIX/B fibres in FoxP1^iSkmTg/Tg^ mice (*Figure* 2N–O), indicating that the leftward shift in the fibre size distribution in FoxP1^iSkmTg/Tg^ mice is primarily driven by the atrophy of larger myofibres. Taken together, these data demonstrate that muscle‐specific FoxP1 over‐expression is sufficient to induce muscle wasting and muscle fibre atrophy.

### Skeletal muscle‐specific induction of FoxP1 induces myopathy

**Figure 3:**
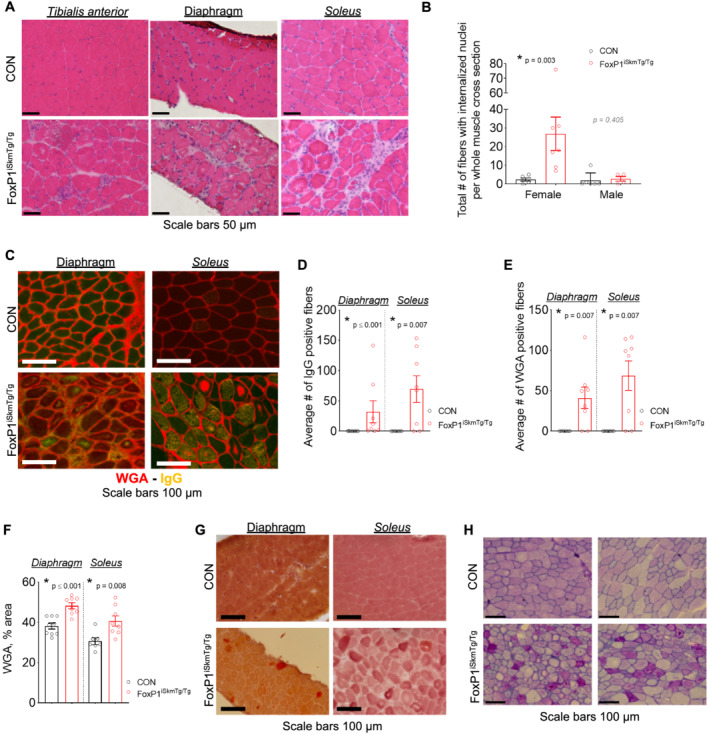
Skeletal muscles of FoxP1^iSkmTg/Tg^ exhibit myopathy. (A) Representative images of haematoxylin and eosin (H&E) stained cross‐sections show increased extracellular space, presence of mononucleated cells, and increased number of myofibres with centralized nuclei in muscles from CRE+ FoxP1^iSkmTg/Tg^ mice compared with CRE‐ littermate controls (CON). (B) Quantification of total number of fibres presenting internalized nuclei in soleus muscle cross‐sections from CRE+ FoxP1^iSkmTg/Tg^ mice and CRE‐ littermate controls. (C) Representative muscle cross‐sections from female CRE+ FoxP1^iSkmTg/Tg^ mice and CRE‐ littermate controls stained with mouse immunoglobulin G (IgG) antibodies and wheat germ agglutinin (WGA). (D–F) muscles from female CRE+ FoxP1^iSkmTg/Tg^ mice show increased presence of intra‐myofibre endogenous IgG (D) and WGA (E) indicative of muscle damage, and increased percent area positive for WGA (F) compared to CRE‐ littermate controls. Data are reported as mean ± SEM. (G) Skeletal muscle cross‐sections from female CRE+ FoxP1^iSkmTg/Tg^ mice stained with Alizarin Red S show dysregulation of intracellular Ca^2+^ compared with CRE‐ littermate controls. (H) Representative images of *tibialis anterior* cross‐sections stained with periodic acid‐Schiff (PAS) reveal the accumulation of PAS + vacuoles in muscles of CRE+ FoxP1^iSkmTg/Tg^ mice compared with CRE‐ littermate controls (CON). Depending on data distribution, unpaired two‐tailed *t*‐tests or Mann–Whitney tests were performed to test for statistical differences between groups. Data are reported as mean ± SEM. Note that data from male CRE+ FoxP1^iSkmTg/Tg^ mice and CRE‐ littermate controls are presented in *Figure* *S6*.

**Figure 4:**
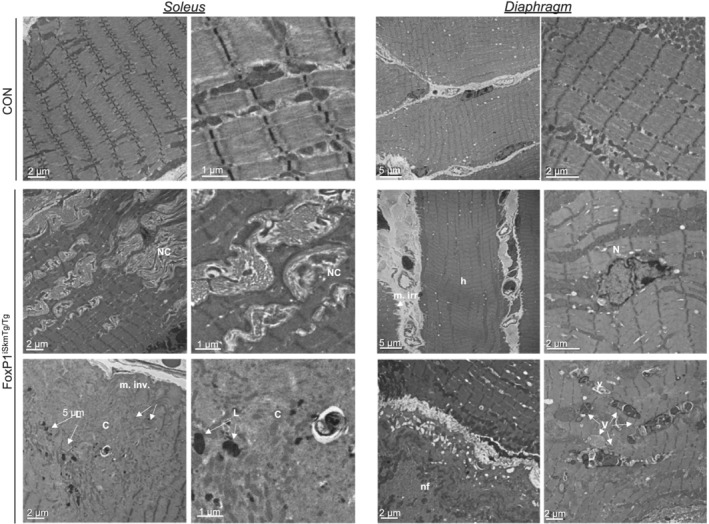
Inducible, skeletal muscle‐specific FoxP1 over‐expression causes ultra‐structural alterations. Transmission electron microscopy images show ultra‐structural alterations in muscles from female CRE+ FoxP1^iSkmTg/Tg^ mice compared with CRE‐ littermate controls. C, core; h, hypercontracted sarcomeres; L, lysosome; m. irr., membrane irregularities; N, internalized nucleus; NC, non‐contractile material; nf, necrotioc fibre; V, vacuoles.

To gain further insights into the effects of FoxP1 up‐regulation in skeletal muscle, we examined skeletal muscle morphology and ultrastructure in multiple muscle groups of FoxP1^iSkmTg/Tg^ mice, including *tibialis anterior*, diaphragm, and *soleus*, which revealed widespread muscle disruptions (*Figures* 3 and S6). In this regard, H&E staining of FoxP1^iSkmTg/Tg^ mice revealed significant myopathy characterized by the presence of centrally nucleated myofibres and increased extracellular space (*Figure* 3A–B), suggesting significant muscle remodelling. This myopathy was also associated with significant myofibre damage, as confirmed through intracellular staining of both WGA and IgG—which requires previous membrane damage^59^, ^60^, ^62^ (*Figures* 3C–E and S6A–C). FoxP1^iSkmTg/Tg^ mice also showed increased total muscle area occupied by WGA—which has been used as a surrogate marker for fibrosis^63^—(*Figures* 3F and S6D), and calcium deposition (*Figure* 3G and *S6E*), which further support the dystrophic‐like changes observed via H&E staining.^63^ We also observed in muscle of FoxP1^iSkmTg/Tg^ mice the presence of various cytosolic abnormalities, including the presence of periodic acid Schiff‐positive vacuoles presumed to be filled with polysaccharides (e.g. glycogen) or other macromolecules containing a high proportion of carbohydrates (*Figure* 3H). These findings were supported via TEM, which also revealed the presence of cellular‐material filled vacuoles (*Figure* 4). Also consistent with our histological findings of muscle damage, TEM revealed marked disruption to sarcomere architecture, and accumulation of non‐contractile material within myofibres of FoxP1^iSkmTg/Tg^ mice.

### Skeletal muscles of FoxP1^iSkmTg/Tg^ mice display muscle weakness

**Figure 5:**
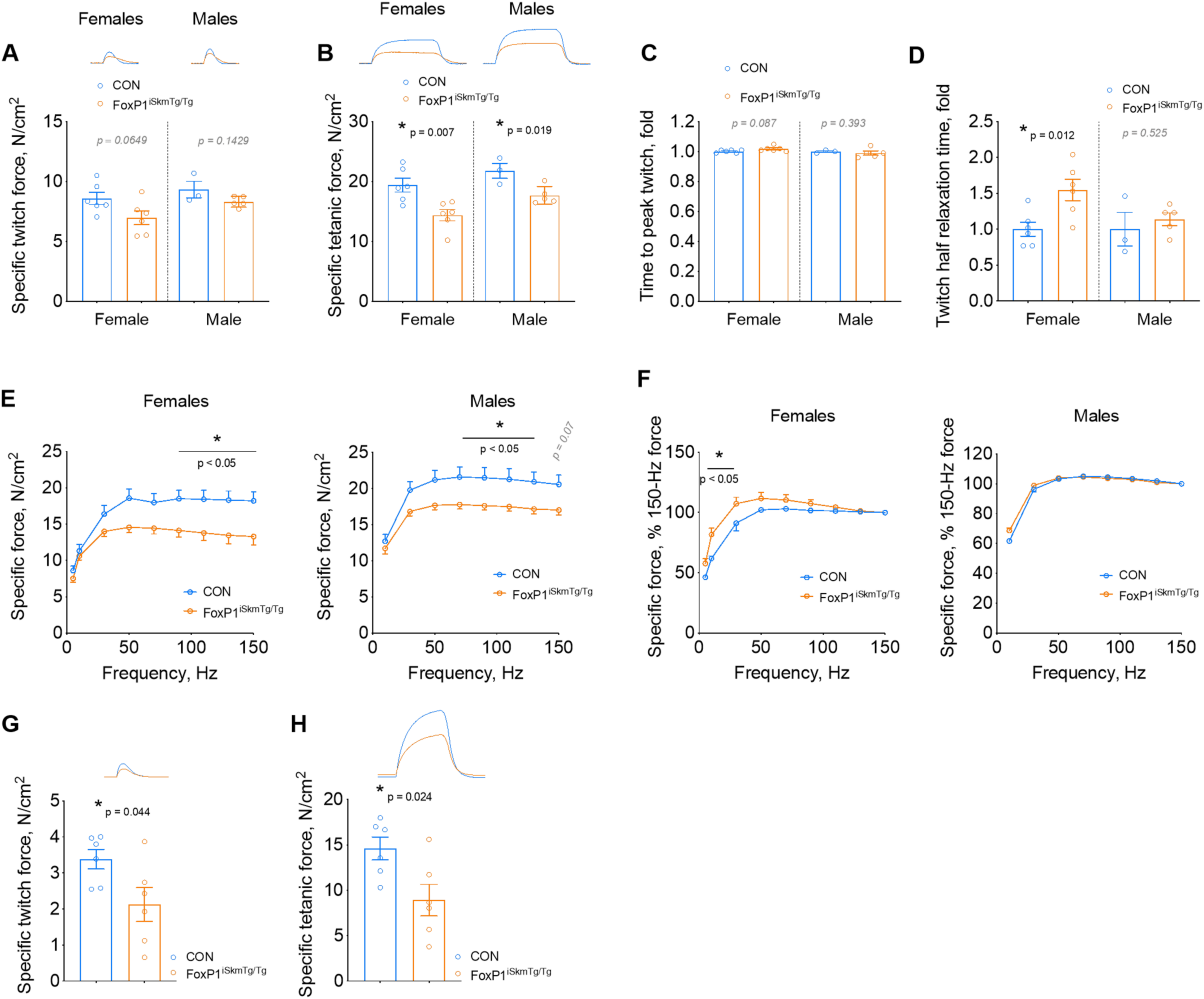
Inducible, skeletal muscle‐specific FoxP1 over‐expression causes skeletal muscle weakness. Specific twitch (A) and tetanic (B) forces recorded from diaphragm strips from CRE+ FoxP1^iSkmTg/Tg^ mice (orange) and CRE‐ littermate controls (blue). Twitch contraction (C) and half relaxation times (D) show slower kinetics in female CRE+ FoxP1^iSkmTg/Tg^ mice vs. CRE‐ littermate controls. (E–F) Force–frequency relationships obtained from CRE+ FoxP1^iSkmTg/Tg^ mice and CRE‐ littermate controls indicate that diaphragms from CRE+ FoxP1^iSkmTg/Tg^ mice show reduced specific forces, and a leftward shift at low stimulation frequencies (F). Twitch (G) and tetanus (H) forces recorded from *soleus* muscles harvested from CRE+ FoxP1^iSkmTg/Tg^ mice (orange) and CRE‐ littermate controls. Depending on data distribution, unpaired two‐tailed *t*‐tests or Mann–Whitney tests were performed to test for statistical differences between groups in panels (A–D) and (G–H). Statistical differences between means were tested by conducting two‐way mixed ANOVAs for panels (E) and (F). Data are reported as mean ± SEM.

On the basis of our findings of both muscle wasting and myopathy in FoxP1^iSkmTg/Tg^ mice, we hypothesized that these mice would also demonstrate muscle weakness. We therefore measured muscle contractile properties in the diaphragm and *soleus* muscles, the latter of which was spared from muscle wasting (*Figure* *S7*), but showed significant myopathy (*Figures* 3, 4 and *S6*). FoxP1^iSkmTg/Tg^ mice showed significantly reduced specific maximal tetanic force produced by muscle strips dissected from female (−26%) and male (−19%) diaphragm, whereas specific twitch force was numerically reduced by 19% in female mice and 11% in male mice, compared with CRE‐ littermate controls (*Figures* 5A–B and *S8*). Noteworthy, diaphragm strips from female FoxP1^iSkmTg/Tg^ mice showed longer half relaxation time (+54%), but unchanged time to peak twitch (+2%) compared with diaphragm strips from CRE‐ littermate controls (*Figure* 5C–D) suggesting that FoxP1 over‐expression resulted in slower contractile properties; to note, this slowing of the twitch properties was not observed in diaphragm strips harvested from male FoxP1^iSkmTg/Tg^ mice (−1% and +15%, respectively). In agreement with the slower contractile properties observed in female mice, a leftward shift in the force–frequency relationship was observed in diaphragm of female mice over‐expressing FoxP1, with higher relative forces produced at 10 and 30 Hz by FoxP1^iSkmTg/Tg^ compared with diaphragm strips from CRE‐ littermates, whereas forces produced at high frequencies were lower (−17% to 27%) in FoxP1^iSkmTg/Tg^ mice compared with CRE‐ littermate controls (*Figure* 5E–F). Similar to the diaphragm, *soleus* muscles from female FoxP1^iSkmTg/Tg^ mice were weaker than *soleus* from CRE‐ littermate controls, with reductions of 19% and 26% in specific twitch and tetanic force, respectively (*Figure* 5G–H). No changes were observed in *soleus* time to peak twitch (+38%) or half relaxation time (+5%) (data not shown). Noteworthy, FoxP1 over‐expression did not affect *soleus* fatigue susceptibility as indicated by the similar extent of force loss measured over the course of a fatiguing protocol (*Figure* *S9*). Taken together, these data indicate that even in skeletal muscles that do not exhibit FoxP1‐induced muscle fibre atrophy, FoxP1 is sufficient to induce significant skeletal muscle weakness.

### FoxP1 exerts transcriptional repression on gene networks involved in skeletal muscle structural development and function, and in cell quality control

**Figure 6:**
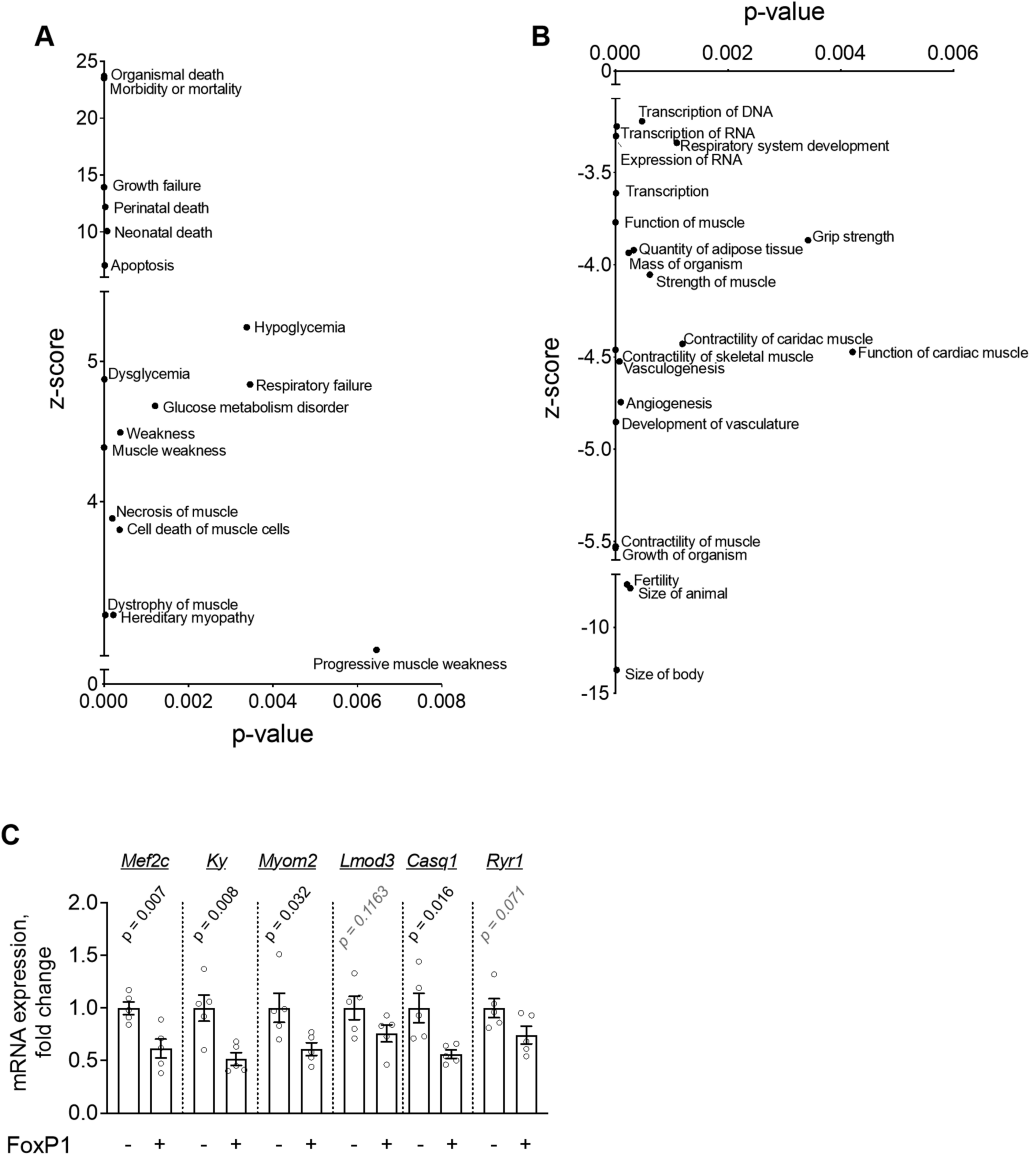
Acute FoxP1 over‐expression in skeletal muscle leads to dysregulation of skeletal muscle homeostatic pathways. Mouse *tibialis anterior* muscles were transfected with a FoxP1 plasmid or empty vector and microarray analyses were conducted on pooled samples (*n* = 3 per group). (A‐B) IPA enriched diseases and functions that are up‐regulated (A) and down‐regulated (B) in response to acute FoxP1 over‐expression. (C) RT‐qPCR validation for selected genes involved in muscle structural development, function, and maintenance. Depending on data distribution, unpaired two‐tailed *t*‐tests or Mann–Whitney tests were performed to test for statistical differences between groups. Data are reported as mean ± SEM.

To gain insights into the early mechanisms underpinning skeletal muscle wasting, weakness, and myopathy induced by FoxP1 over‐expression, mouse *tibialis anterior* were transfected with a FoxP1 plasmid or empty vector and muscle tissues harvested 4 days later for transcriptomic analysis. Muscle samples within each group were pooled, and microarray analysis conducted to identify genes differentially regulated by FOXP1. The top 50 most enriched terms obtained for Biological Process, Molecular Function, Cellular Component, Kyoto Encyclopedia of Genes and Genomes, and Reactome gene ontology are presented in *Data S1*. For each category, the 50 most enriched terms were all predicted to be down‐regulated—which is in line with the known function of FoxP1 as a transcriptional repressor.^21^, ^22^, ^23^ Noteworthy, among the most significantly enriched annotations *down‐regulated* by FoxP1 were those related to skeletal muscle structure, function, and development (sarcomere, false discovery rate (FDR) = 2.90E‐15; sarcoplasmic reticulum, FDR = 2.28E‐08; myogenesis FDR = 1.25E‐05), chromatin remodelling through histone acetylation (histone acetyltransferase complex, FDR = 8.78E‐14; histone H4‐K16 acetylation, FDR = 1.09E‐05), cell quality control (autophagy‐animal, FDR = 2.09E‐19; phagophore assembly site, FDR = 2.56E‐06), and cell metabolism (glycogenolysis, FDR = 4.1E‐04) (*Data S1*). Additional insight into the potential downstream consequences of these transcriptional perturbations was further obtained using the diseases and functions analyses provided by Ingenuity Pathway Analysis (*Figure* 6A), which predicted terms related to organismal death, growth failure, and muscle cell death to be up‐regulated by FoxP1. In agreement with this, functions related to body size and muscle contractility were predicted to be down‐regulated in muscles over‐expressing FoxP1 (*Figure* 6B). On the basis of these findings, we validated several target genes critical to muscle structural development, function and maintenance as downstream target genes repressed by FoxP1 using RT‐qPCR, including myocyte enhancer factor 2c (*Mef2c*), kyphoscoliosis peptidase (*Ky*), myomesin 2 (Myom2), leiomodin 3 (*Lmod3*), calsequestrin 1 (*Casq1*), and ryanodine receptor 1 (*Ryr1*, *Figure* 6C). Taken together, these transcriptional data align with our histological findings of muscle wasting, myopathy, and weakness in muscles of FoxP1^iSkmTg/Tg^ mice and implicate the repression of muscle‐specific genes that regulate muscle structure and function as potential underlying mechanisms.

### FoxP1 negatively regulates muscle fibre regrowth following cardiotoxin injury

**Figure 7:**
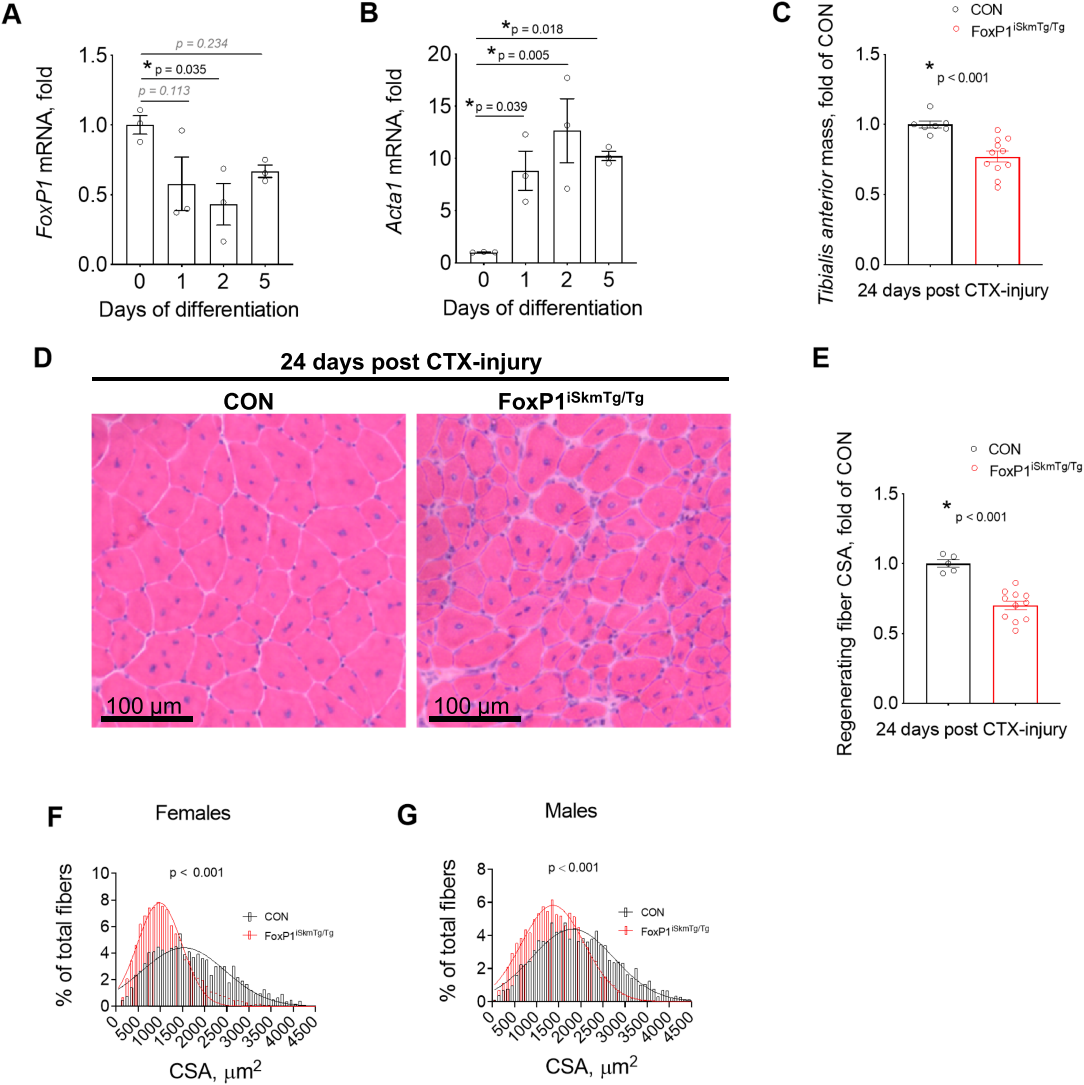
Inducible, skeletal muscle‐specific up‐regulation of FoxP1 impedes muscle regeneration following injury. (A–B) *FoxP1* (A) and *Acta1* (B) mRNA expression measured in proliferating C2C12 myoblasts (Day 0) and following 1, 2, or 5 days of differentiation into myotubes. (C–G) *Tibialis anterior* muscles of CRE+ FoxP1^iSkmTg/Tg^ mice and CRE‐ littermate controls (CON) were injured via direct injection of cardiotoxin on the same day as tamoxifen treatments were initiated. Twenty‐four days post‐injury, FoxP1^iSkmTg/Tg^ mice showed reduced muscle mass (C), and reduced cross‐sectional area (CSA) of regenerating myofibres, as visualized in *tibialis anterior* muscle cross‐sections stained with H&E (D) and as quantified in (E–G). Cross‐sectional area data were binned, fit with a Gaussian least squares regression and significance was determined by calculating the extra sum‐of‐squares *F* test. Statistical differences were determined by conducting one‐way ANOVAs in panels (A) and (B), and unpaired two‐tailed *t*‐tests or Mann–Whitney tests in panels (C) and (E). Data are reported as mean ± SEM.

On the basis of our finding that FoxP1 repressed numerous muscle‐specific genes known to be critical for muscle cell differentiation and maturation into functional muscle fibres, we examined the mRNA levels of *FoxP1* in C2C12 cells undergoing myogenic differentiation. We found that the mRNA levels of *FoxP1* are highest in proliferating C2C12 myoblasts and decline during differentiation into mature myotubes (*Figure* 7A). These data align with published microarray data,^64^ and taken together with our findings in FoxP1^iSkmTg/Tg^ mice, suggest that FoxP1 could function as a negative regulator of muscle differentiation. We therefore determined *in vivo,* whether FoxP1^iSkmTg/Tg^ mice show disruptions in *de novo* myofibre regeneration and regrowth following cardiotoxin injury. *Tibialis anterior* muscles of Foxp1^iSkmTg/Tg^ mice and CRE‐ littermate controls were therefore injected with cardiotoxin, and on the same day, we initiated tamoxifen treatments—consisting of daily tamoxifen injections for 5 days, followed by maintenance on a tamoxifen diet until Day 24, when muscle tissues were harvested for morphological analyses. Cardiotoxin injury causes complete degeneration and removal of adult muscle fibres, followed by proliferation of skeletal muscle stem and myogenic precursor cells that subsequently undergo differentiation, fuse and regrow into fully functional *de novo* myofibres. In Foxp1^iSkmTg/Tg^ mice, all myogenic precursors undergoing differentiation would be expected to undergo CRE‐mediated recombination and up‐regulate FoxP1 as soon as the skeletal muscle actin (*Acta1*) promoter, which drives CRE expression, becomes active. In C2C12 muscle cells, we show that *Acta1* mRNA is elevated as early as 1 day post‐differentiation ( approximately nine‐fold, *Figure* 7B). Upon tissue harvest 24 days post‐injury, injured muscles from FoxP1^iSkmTg/Tg^ mice weighed ~21% less than injured muscles from CRE‐ controls (*Figure* 7C), and displayed significantly smaller (32%) regenerating fibres (*Figure* 7D–G).

### FoxP1‐dependent muscle wasting requires histone deacetylase activity

**Figure 8:**
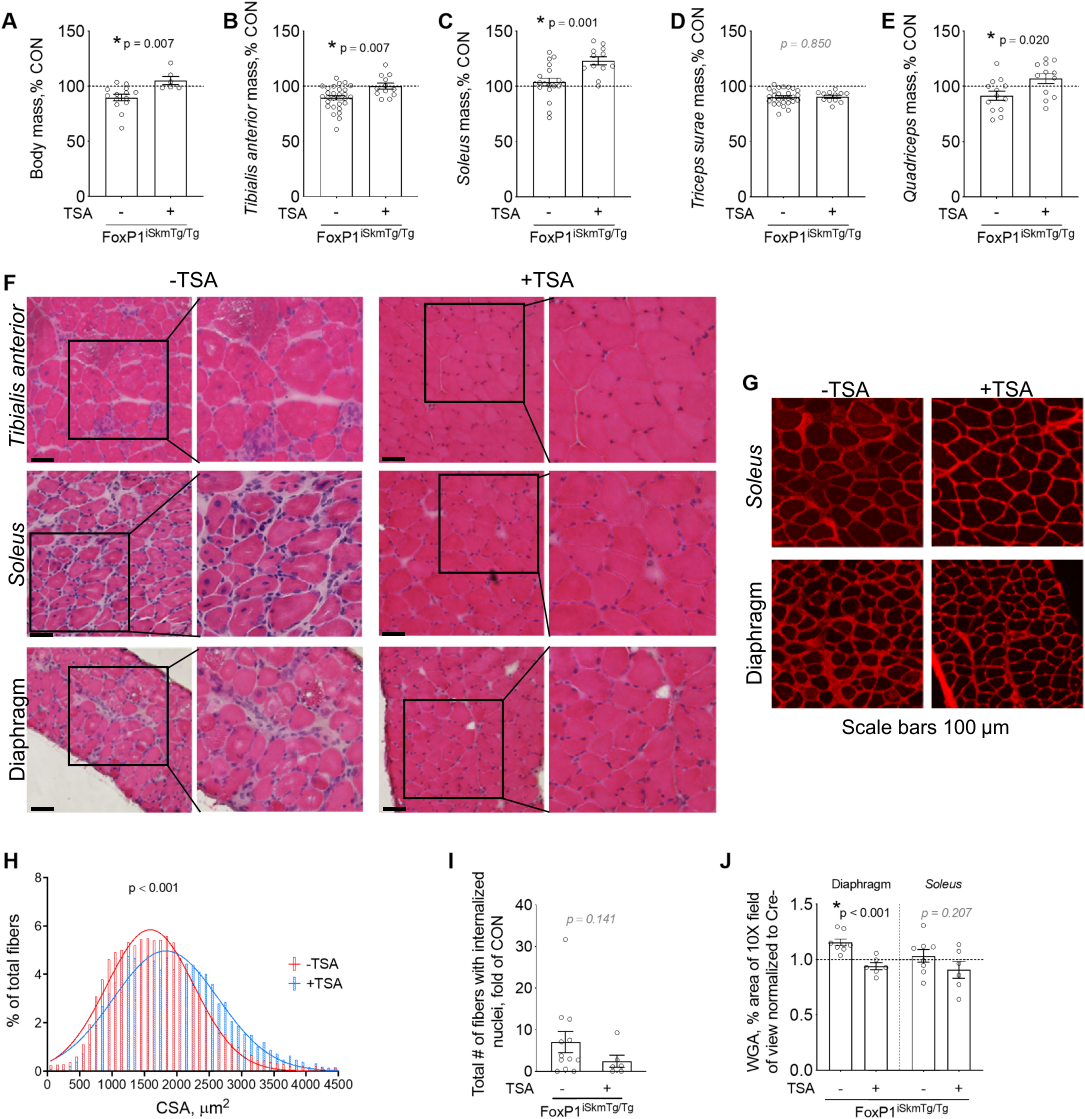
FoxP1‐dependent induction of body and skeletal muscle wasting occurs through an HDAC‐dependent manner. (A–I) Tamoxifen‐treated CRE+ FoxP1^iSkmTg/Tg^ mice received daily intraperitoneal (IP) injections of trichostatin A (TSA, a pan HDAC inhibitor). TSA treatment blunted the FoxP1‐dependent loss of body mass (A) and skeletal muscle mass (B–E). Body and muscle masses are expressed as percent of sex‐matched CRE‐ littermate controls (CON). (F–G) Representative images of FoxP1^iSkmTg/Tg^ muscle cross‐sections stained with haematoxylin & eosin (H&E, F) and wheat germ agglutinin (WGA, G) show that TSA treatment protects against FoxP1‐induced muscle fibre atrophy (H), myopathy including degeneration/regeneration (I), and extracellular matrix (ECM) deposition (J). Data are normalized to CRE‐ littermate controls and reported as mean ± SEM. Cross‐sectional area data were binned, fit with a Gaussian least squares regression and significance was determined by calculating the extra sum‐of‐squares *F* test. Depending on data distribution, unpaired two‐tailed *t*‐tests or Mann–Whitney tests were performed to test for statistical differences between groups in panels (A–E) and (I and J). [Note that data for mice not treated with TSA originate from the same mice as in *Figures* 2, 3, *S3*, and *S6*].

Thus far, we have established that FoxP1 acts as a transcriptional repressor of skeletal muscle gene expression whose up‐regulation in skeletal muscle is sufficient to cause muscle damage and wasting and impede regrowth of regenerating myofibres. In other cell types, FoxP1 has been established to exert transcriptional repression and mediate cellular functions through its interaction with transcriptional repressor complexes containing histone deacetylase (HDAC) proteins.^21^ We therefore further determined whether HDACs are necessary for FoxP1 to induce skeletal muscle wasting and myopathy. As shown in *Figure* 8A–E, daily treatment of FoxP1^iSkmTg/Tg^ mice with TSA completely prevented FoxP1 from inducing both body and skeletal muscle wasting, markedly improved muscle morphology (*Figure* 8F–G), and prevented fibre atrophy (*Figure* 8H). Moreover, skeletal muscles from FoxP1^iSkmTg/Tg^ mice treated with TSA were devoid of vacuoles (*Figure* 8F), showed numerical reduction in the presence of internalized nuclei (*Figure* 8I), and did not exhibit increased extracellular matrix expansion (*Figure* 8J). TSA treatment of mice in which FoxP1 over‐expression was acutely induced in *tibialis anterior* by transfection of FoxP1 expression plasmid also disrupted the ability of FoxP1 to exert transcriptional repression of several muscle‐specific genes (*Figure S10*). Together, these data thus implicate HDACs as key mediators of the skeletal muscle phenotype induced by FoxP1, including the repression of muscle‐specific genes and its induction of skeletal muscle damage, wasting, and remodelling.

### FoxP1 represses MEF2 target gene transcription during cancer cachexia

**Figure 9:**
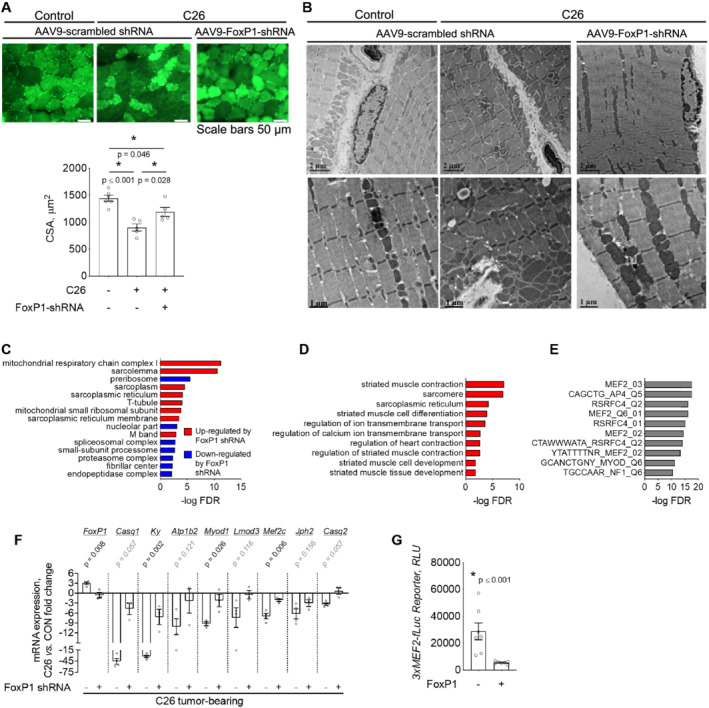
FoxP1 mediates cancer‐induced muscle atrophy and the repression of MEF2 target genes. (A) Transduction of *tibialis anterior* muscles of C26‐tumour bearing mice with an AAV9 vector encoding FoxP1‐shRNA blunts the C26‐induced reduction in muscle fibre cross‐sectional area (CSA) that is observed in muscles transduced with AAV9 encoding scrambled‐shRNA. (B) Representative transmission electron microscopy images of *tibialis anterior* muscles from sham and C26‐tumour bearing mice transduced with the AAV9 encoding FoxP1 shRNA or scrambled shRNA, showing ultrastructural alterations in response to C26 tumour burden, that are improved in response to FoxP1 knockdown. (C) Microarray analyses conducted on pooled samples (*n* = 3 per group) and further analysed with string to identify enriched cellular components, indicate that FoxP1 knockdown attenuates the tumour‐induced repression of genes involved in muscle structure/function (blue categories) and reduces the activation of genes involved in protein breakdown (red categories). (D–E) Genes repressed by two‐fold or more in response to C26 tumour burden that were rescued by two‐fold or more in muscles expressing FoxP1‐shRNA (249 genes) were further analysed using DAVID to identify enriched non‐redundant functional annotations (D), and using gene set enrichment analysis (GSEA) and the molecular signatures database to identify enriched transcription factor binding motifs among gene promoters (E). FoxP1 targets repressed in muscle of C26 mice were enriched for MEF2 and MYOD target genes involved in muscle contraction and muscle cell differentiation. (F) RT‐qPCR validation demonstrating that FoxP1 knockdown rescues the C26‐induced repression of several MEF2 target genes involved in muscle structural development, function, and maintenance. (G) Mouse *tibialis anterior* muscles were transfected with a FoxP1 expression plasmid or empty vector, plus a MEF2‐dependent luciferase reporter to assess MEF2 activity. RLU = relative light unit. Statistical differences were determined by conducting a one‐way ANOVA in panel (A), unpaired two‐tailed *t*‐test or Mann–Whitney tests depending on data distribution in panel (F) and Wilcoxon test in panel (G). Data are reported as mean ± SEM.

On the basis of our findings that FoxP1 is sufficient to repress muscle‐specific gene expression and induce muscle wasting, we next sought to determine whether the up‐regulation of endogenous FoxP1 mediates muscle wasting in response to tumour burden. Mouse *tibialis anterior* muscles were therefore injected with AAV9 encoding FoxP1 shRNA or scrambled shRNA 2 weeks before C26 tumour inoculation and tissues were harvested at Institutional Animal Care and Use Committees‐mandated endpoint. Knockdown of FoxP1 partially attenuated muscle fibre atrophy induced by tumour burden (*Figure* 9A) and visually improved muscle ultrastructure as observed via TEM (*Figure* 9B). Microarray analysis on *tibialis anterior* pooled samples revealed FoxP1 as a key mediator of the cancer‐induced transcriptional repression of gene networks critical to muscle structure, function, and regeneration/repair. These findings were supported through both String analysis of all genes (*Figure* 9C), and through follow‐up analysis using DAVID, of the 249 genes identified to be repressed by two‐fold or more in response to C26, whose expression was rescued by FoxP1‐shRNA by two‐fold or greater (*Figure* 9D). Indeed, functional annotations related to skeletal muscle contraction, muscle cell differentiation, and calcium ion transmembrane transport were highly enriched. We further analysed these 249 FoxP1 targets repressed in response to tumour burden using Gene Set Enrichment Analysis and the Molecular Signatures Database to identify conserved transcription factor binding motifs commonly shared among their gene promoters (*Figure* 9E). Included among the top 10 most highly enriched binding motifs were those corresponding to the myogenic transcription factors, myocyte enhancer factor 2 (MEF2), and myogenic differentiation 1 (MYOD1), which cooperatively regulate muscle‐specific gene expression.^65^, ^66^, ^67^ Indeed, several downstream targets of MEF2 involved in muscle structure and function were among the top FoxP1 target genes repressed in response to tumour burden (*Data S2*), including *Casq1*, *Casq2*, *Lmod3*, *Ky*, and *Mef2c* (*Figure* 9F)—suggesting that FoxP1 up‐regulation disrupts MEF2 transcriptional activity. To test this, we acutely transfected mouse muscle with a Foxp1 expression plasmid plus a MEF2‐dependent luciferase reporter using plasmid injection and electroporation and found that FoxP1 significantly repressed the MEF2 reporter (*Figure* 9G). These findings collectively highlight FoxP1 as a negative regulator of MEF2, whose up‐regulation in response to tumour burden contributes to the repression of MEF2 target gene transcription, ultrastructural alterations and to the normal atrophy process.

### FoxP1 is increased in skeletal muscle of cachectic cancer patients

**Figure 10:**
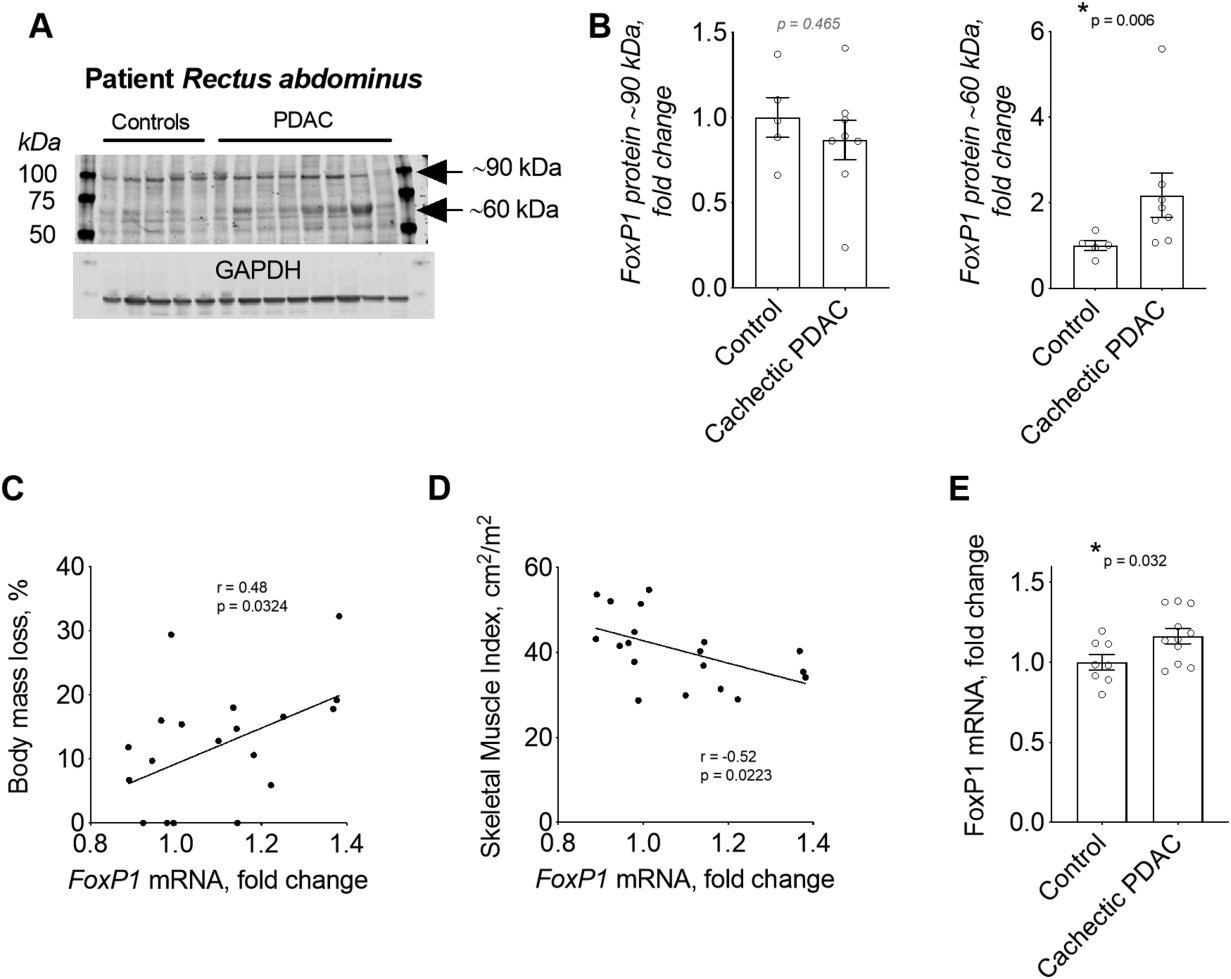
FoxP1 expression is increased in skeletal muscle of patients with pancreatic ductal adenocarcinoma (PDAC) exhibiting cachexia. (A–B) FOXP1 protein expression is increased in *rectus abdominis* muscle biopsies from cachectic PDAC patients compared with non‐cancer control patients. In panel (A), arrows point to the two FoxP1 isoforms quantified in B. (C–E) *FOXP1* mRNA expression in *rectus abdominis* muscle of PDAC patients correlates positively with body mass loss (C) and negatively with skeletal muscle index (D) and is increased in cachectic PDAC patients when compared with weight‐stable non‐cancer controls with normal muscularity (E). Note: mRNA data were extracted from microarray analyses previously analysed and published in combination with histological assessment of muscle biopsies^20^ and in combination with MRI‐based measures of skeletal muscularity and muscle quality.^68^ Statistical differences were determined by conducting unpaired two‐tailed *t*‐test or Mann–Whitney tests depending on data distribution in panels (B) and (E), and simple linear regression analyses in panels (C) and (D). Data are reported as mean ± SEM.

On the basis of our findings that FoxP1 is both sufficient and required for cancer‐associated muscle wasting, we sought to determine whether our findings are relevant to skeletal muscle wasting in human cancer patients. We thus measured the protein expression of FOXP1 in muscle biopsies from non‐cancer controls and patients with PDAC—a cancer type displaying one of the highest incidences of cachexia.^7^ Compared with non‐cancer controls, FOXP1 protein expression was increased in skeletal muscle of cancer patients defined as cachectic based on body mass loss of >5% in combination with muscle depletion (i.e. low skeletal muscle index) (*Figure* 10A–B). Moreover, extraction of skeletal muscle microarray data from a larger cohort of PDAC patients^20^, ^68^ further revealed a positive correlation between the mRNA levels of *FOXP1* and body mass loss (*Figure* 10C), and a negative correlation between the mRNA levels of *FOXP1* and skeletal muscle index (*Figure* 10D). In support of these findings, *FOXP1* mRNA levels were significantly higher in PDAC patients classified as cachectic compared with weight‐stable non‐cancer controls (*Figure* 10E), which aligns with our finding of increased abundance of FOXP1 protein. These data provide significant translational relevance to our findings in mouse models, which demonstrate that FoxP1 is sufficient to induce muscle wasting and weakness, and mediates the normal muscle atrophy response to tumour burden.

## Discussion

The present study identifies FoxP1 as a novel transcriptional repressor in skeletal muscle, whose conditional activation in adult muscle fibres is sufficient to induce muscle wasting and dysfunction, and impedes the regrowth of regenerating myofibres. We demonstrate that FoxP1‐induced muscle wasting and remodelling is mediated through HDACs—which are known to interact with FoxP1 as part of larger transcriptional complexes—to induce gene repression. We further show that these findings are relevant to muscle wasting associated with cancer, as FoxP1 expression was elevated in multiple mouse models of cancer cachexia, and its knockdown attenuated cancer‐associated muscle fibre atrophy. Using skeletal muscle biopsies from cancer patients, we demonstrate that these findings are relevant to human cancer cachexia, because the levels of FOXP1 mRNA and protein were elevated in patients defined as cachectic.

Previous studies from our laboratory,^13^, ^15^ and others,^69^ have established the importance of the FoxO transcription factors in promoting tumour‐induced muscle wasting, and our previous work identified FoxP1—whose role in skeletal muscle has not been studied to this date—as a FoxO‐dependent gene.^15^ Our present data extend this previous work by showing that skeletal muscle expression of FoxP1 is increased in multiple models of cancer cachexia in which FoxO factors are also elevated, and by further identifying FoxP1 as a downstream target of FoxO1. This finding aligns with microarray data collected from mice conditionally deleted for *FoxO1*/*FoxO3a*/*FoxO4* specifically in skeletal muscle, in which *FoxP1* was identified as a FoxO‐target in response to starvation.^70^ Importantly, FoxO1 is consistently identified as a cachexia‐associated gene increased in muscle of cancer patients,^19^, ^20^ and we show that increased levels of FoxP1 are also associated with cachexia in cancer patients. Thus, cancer‐induced muscle wasting could be mediated, at least in part, through a FoxO1–FoxP1‐dependent mechanism. In support of this, over‐expression of FoxP1 in skeletal muscles led not only to skeletal muscle wasting but also body wasting and was often coincident with fat wasting, which are all features of cachexia observed in cancer patients and tumour‐bearing mice. Moreover, Foxp1^iSkmTg/Tg^ mice also showed evidence of additional myopathies similar to that observed in skeletal muscle biopsies from cachectic cancer patients,^20^ including evidence of damage, myofibres with centralized nuclei, and expansion of extracellular matrix—the latter of which could be related to our finding that FoxP1 up‐regulation also disrupted the muscle regenerative process.^71^

Genome‐wide transcriptomic analyses in muscles acutely over‐expressing FoxP1 revealed FoxP1 as a potent transcriptional repressor of skeletal muscle gene expression—with significant enrichment of functional annotations related to skeletal muscle structure, function, and myogenesis. In line with this, Foxp1^iSkmTg/Tg^ mice showed significant disruptions in *de novo* myofibre regrowth following cardiotoxin injury, which relies on the activation of muscle‐specific genes to complete the regenerative process.^67^ These findings were also congruent with the role of endogenous FoxP1 in a mouse model of cancer cachexia, whose knockdown blocked the cancer‐induced repression of genes critical to muscle structure, function, and muscle differentiation. Importantly, promoter analysis of target genes repressed by FoxP1 during cancer revealed strong enrichment of binding sites for MEF2—a family of transcription factors that activate muscle‐specific genes and are together required for muscle differentiation and regeneration.^67^ In further support of FoxP1 as a negative regulator of MEF2, we found that FoxP1 was also sufficient to repress a MEF2‐dependent luciferase reporter. These findings are noteworthy, because we recently showed that MEF2c gain‐of‐function protects against cancer‐associated muscle wasting and weakness,^68^ and highlight FoxP1 up‐regulation as a novel mechanism that contributes to the disruption of MEF2 target gene transcription during cancer cachexia.

Mechanistically, our findings indicate that HDACs are necessary for FoxP1 to elicit skeletal muscle wasting and play a role in FoxP1‐dependent transcriptional repression. Indeed, treatment of Foxp1^iSkmTg/Tg^ mice with TSA completely prevented the muscle wasting phenotype induced in these mice and interfered with FoxP1‐dependent repression of several muscle‐specific genes. These findings align with published studies that have both established HDACs as necessary mediators of FoxP1 cellular functions^21^ and identified FoxP1 to induce gene repression through its interaction and recruitment of transcriptional repressor complexes containing HDAC proteins, including the Nucleasome Remodelling and deacetylation complex,^21^ and nuclear receptor corepressor 2 (also known as SMRT^23^) to gene promoters. It is therefore possible that FoxP1 may exert transcriptional repression of skeletal muscle target genes through a similar mechanism. In fact, muscle‐specific gene transcription is tightly controlled through the opposing roles of histone acetyltransferases and HDAC proteins, with HDAC activity and the associated NCOR complex repressing gene transcription.^72^ However, it is critical to acknowledge that HDACs regulate numerous transcription factors, whose dysregulation in the presence of TSA could indirectly interfere with FoxP1 functions in skeletal muscle. Thus, additional studies are needed to not only identify direct FoxP1 targets, but to determine whether FoxP1 occupancy of target gene promoters impacts the occupancy of HDACs, and other regulatory factors that together dictate promoter activity and promoter accessibility.

While the current study demonstrates the biological significance of full‐length FoxP1 in mouse skeletal muscle, which is transcribed by the FoxP1A transcript, both mice and humans express several additional truncated FoxP1 isoforms that result from alternative splicing.^61^, ^73^ In fact, several truncated versions of FoxP1 were observed through western blots of skeletal muscle from mice and humans, with a smaller FoxP1 isoform between ~55 and 65 kDa showing increased abundance in response to cancer cachexia. Thus, future studies are needed to not only delineate the predominant FoxP1 isoforms expressed in skeletal muscle under baseline conditions and in response to cancer cachexia but to also determine their functional importance in muscle.

In summary, our data identify FoxP1 as a novel transcriptional repressor of skeletal muscle gene expression that is up‐regulated in multiple models of cancer cachexia and in muscle of cachectic cancer patients that is sufficient to induce features of cachexia, including body mass loss, muscle wasting and weakness, and impaired muscle regeneration. Our data further indicate that the muscle wasting phenotype induced by FoxP1 is mediated through an HDAC‐dependent mechanism, and that similar to treatment with HDAC inhibitors,^74^ knockdown of FoxP1 also confers partial protection against cancer‐induced muscle fibre atrophy. These findings therefore indicate that blocking the activity of specific transcriptional repressor complexes that regulate muscle‐specific targets could be effective strategies to impede cancer‐associated muscle wasting and weakness.

## Ethics statement

The authors of this manuscript certify that they comply with the ethical guidelines for authorship and publishing in the Journal of Cachexia, Sarcopenia and Muscle.

## Author contributions

SMJ and ARJ conceived the study; DN, RLN, CSC, SMJ, and ARJ participated in the data acquisition, analysis, and interpretation; JGT collected the human samples; HH created mice with conditional transgenic expression of FoxP1 used to generate FoxP1^iSkmTg/Tg^ mice; DN and SMJ wrote the manuscript. All authors read and approved the submitted version and have agreed to be personally accountable for the author's own contributions and to ensure that questions related to the accuracy or integrity of any part of the work are appropriately investigated and resolved.

## Funding

This work was supported by the National Institute of Arthritis, Musculoskeletal and Skin Diseases (R01AR060209 to ARJ). D.N. was supported by the Swiss National Science Foundation (P400PM_180814), and R.L.N. was supported by a National Institute of Child Health and Human Development Grant (T32‐HD‐043730).

## Conflict of interest

The authors declare that they have no conflict of interest.

## Supporting information


**Data S1.** Supporting Information

**Data S2.** Supporting Information

**Figure S1–S10.** Transfection of a FoxP1 plasmid induces fiber atrophy. Tibialis anterior muscles co‐transfected with a GFP empty vector and either a FoxP1 expression plasmid or empty vector show reduced (A) muscle mass and (B) fiber cross‐sectional area (CSA). Minimum feret diameters (MFD) were binned, fit with a Gaussian least squares regression and significance was determined by calculating the extra sum‐of‐squares F test. Data are shown as Mean ±SEM.
