# Supplementary material for: FoxP1 is a transcriptional repressor associated with cancer cachexia that induces skeletal muscle wasting and weakness
Source: J Cachexia Sarcopenia Muscle. 2021 Feb 1;12(2):421–42. doi: 10.1002/jcsm.12666 (PMC8061399; doi:10.1002/jcsm.12666)
Supplement: Supplementary file 3 — Figure S1–S10. Transfection of a FoxP1 plasmid induces fiber atrophy. Tibialis anterior muscles co‐transfected with a GFP empty vector and either a FoxP1 expression plasmid or empty vector show reduced (A) muscle mass and (B) fiber cross‐sectional area (CSA). Minimum feret diameters (MFD) were binned, fit with a Gaussian least squares regression and significance was determined by calculating the extra sum‐of‐squares F test. Data are shown as Mean ±SEM. [file JCSM-12-421-s001.pdf]

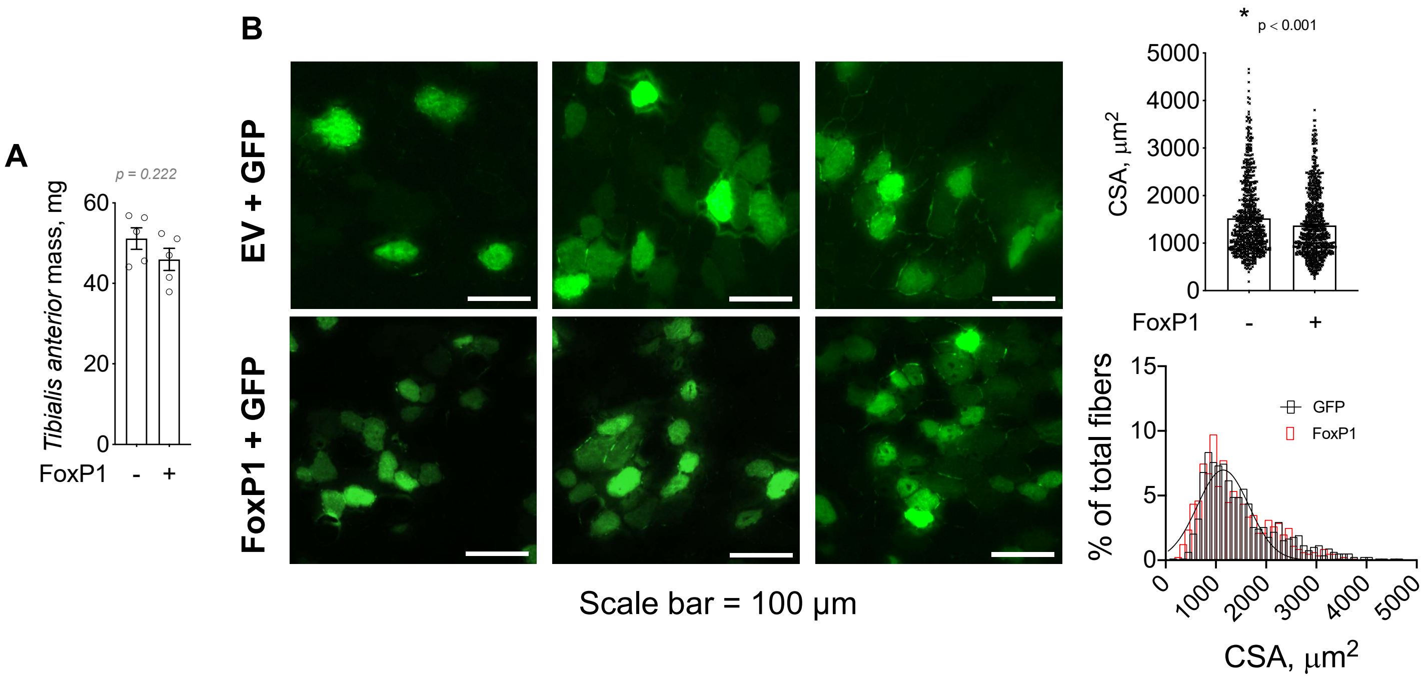

Figure S1. Transfection of a FoxP1 plasmid induces fiber atrophy. *Tibialis anterior* muscles co-transfected with a GFP empty vector and either a FoxP1 expression plasmid or empty vector show reduced (**A**) muscle mass and (**B**) fiber cross-sectional area (CSA). Minimum feret diameters (MFD) were binned, fit with a Gaussian least squares regression and significance was determined by calculating the extra sum-of-squares F test. Data are shown as Mean ± SEM.

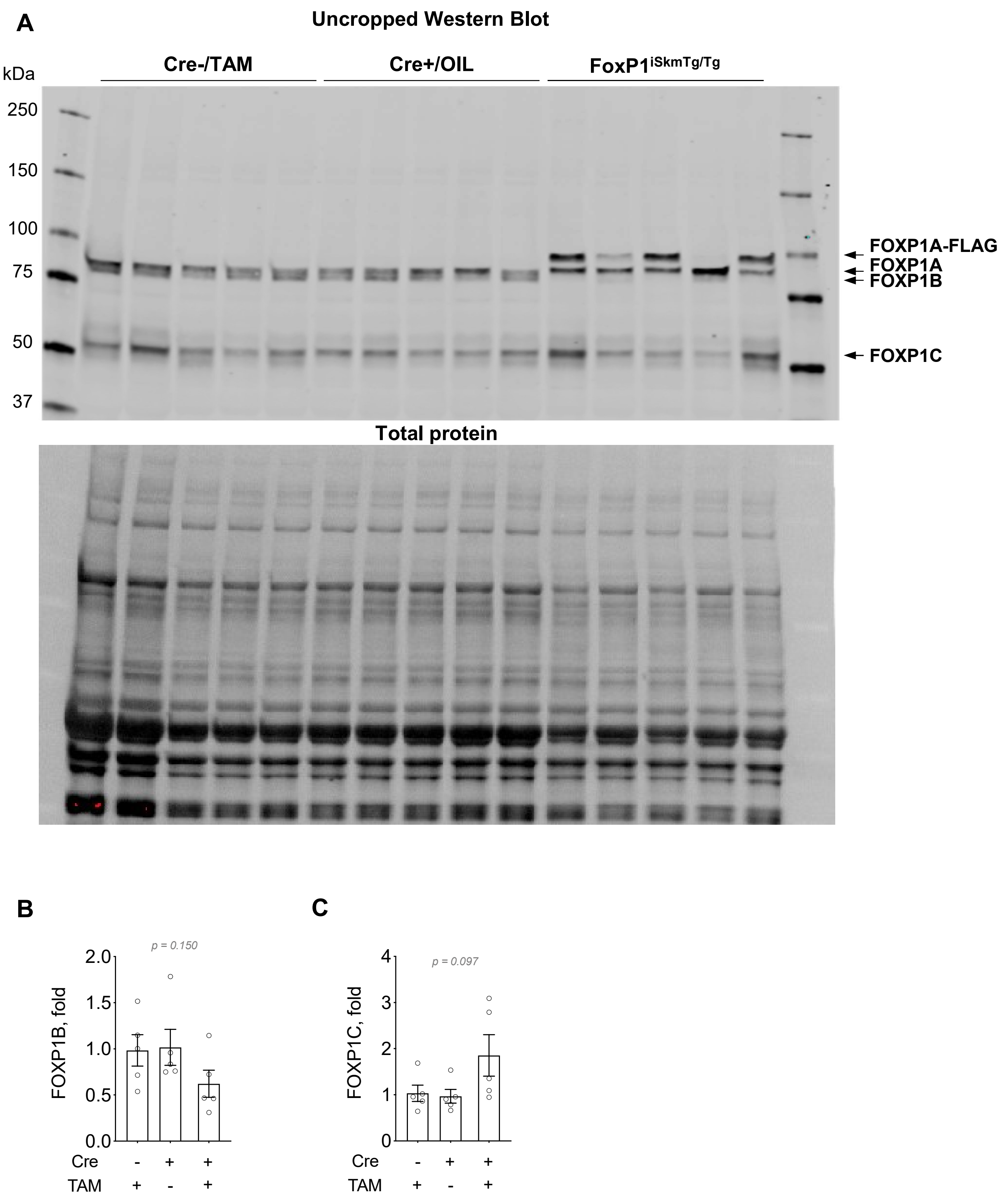

Figure S2. Tamoxifen treatment increases FoxP1A expression in FoxP1$^{iSkmTg/Tg}$ *triceps surae* muscles. (**A**) Uncropped images of the western blot presented in Fig. 2B. (**B-C**) Quantification of FOXP1B (**B**) and FOXP1 C (**C**) expression in CRE+ FoxP1$^{iSkmTg/Tg}$, and CRE- littermate and genetic controls, normalized to total protein. Data are shown as Mean ± SEM.

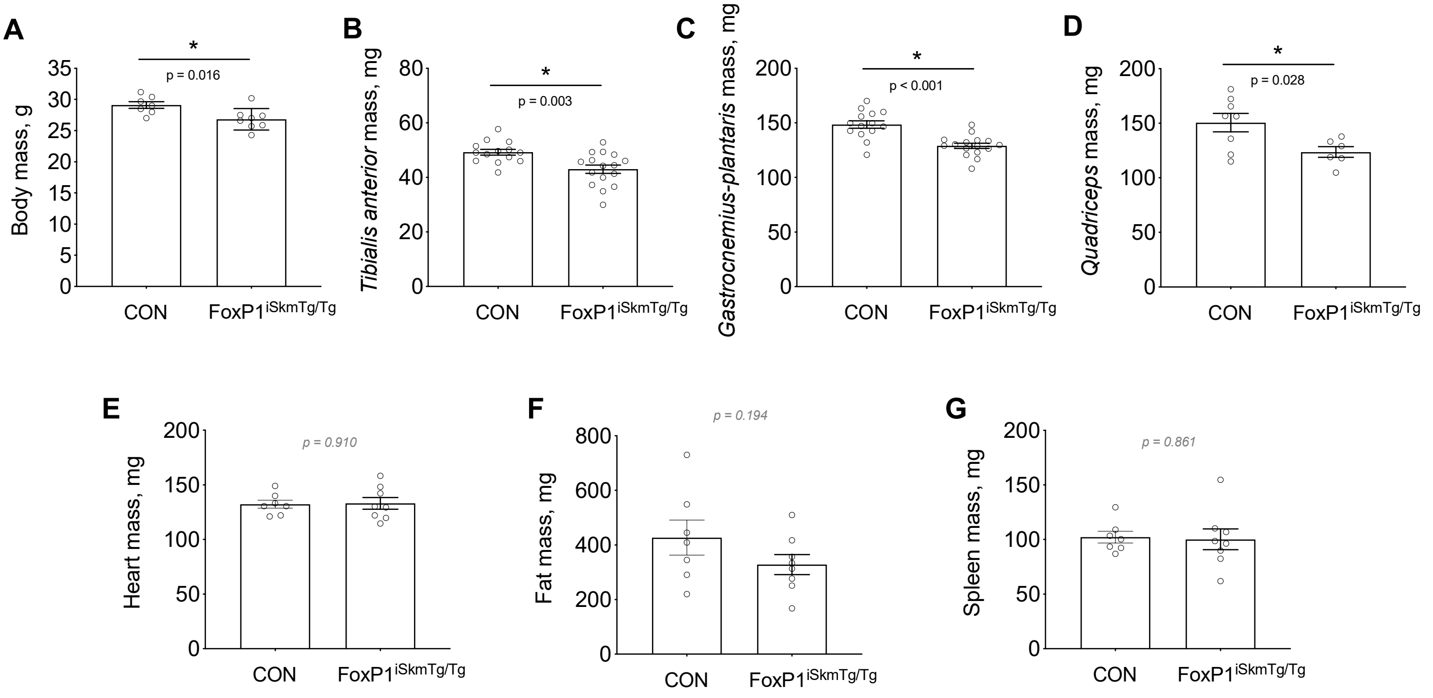

Figure S3. Skeletal muscle-specific over-expression of FoxP1 leads to muscle wasting in *male* mice. Tamoxifen treatment (started at ≥ 9 weeks of age) induces body wasting (**A**) and skeletal muscle wasting (**B-D**) but not heart (**E**) or fat (**F**) wasting. (**G**) Skeletal muscle-specific over-expression of FoxP1 does not affect spleen mass. CON = CRE- littermates. Data are shown as Mean ± SEM.

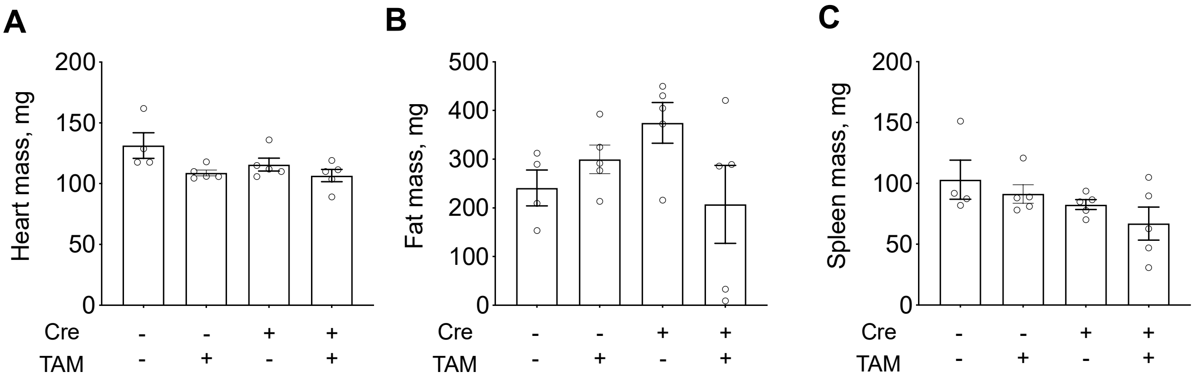

Figure S4. Skeletal muscle-specific over-expression of FoxP1 does not induce changes in (A) heart, (B) gonadal fat and (C) spleen masses in female mice. Note, however that several FoxP1$^{iSkmTg/Tg}$ mice showed fat wasting. Data are reported as mean ± SEM.

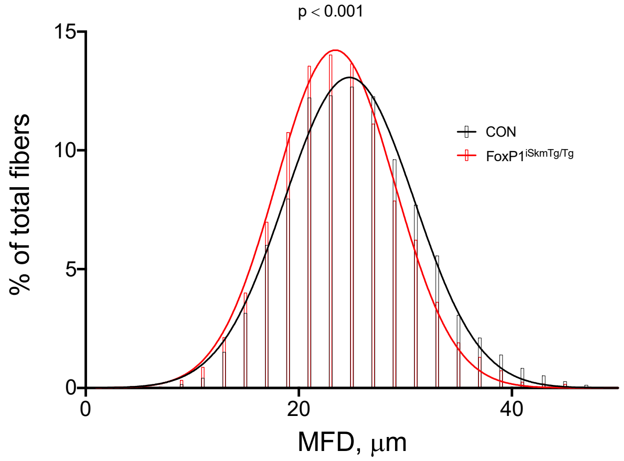

Figure S5. Skeletal muscle-specific over-expression of FoxP1 induces diaphragm fiber atrophy as indicated by the leftward shift observed in fiber size distribution in FoxP1$^{iSkmTg/Tg}$ mice *vs.* genetic controls (CON). Minimum feret diameters (MFD) were binned, fit with a Gaussian least squares regression and significance was determined by calculating the extra sum-of-squares F test.

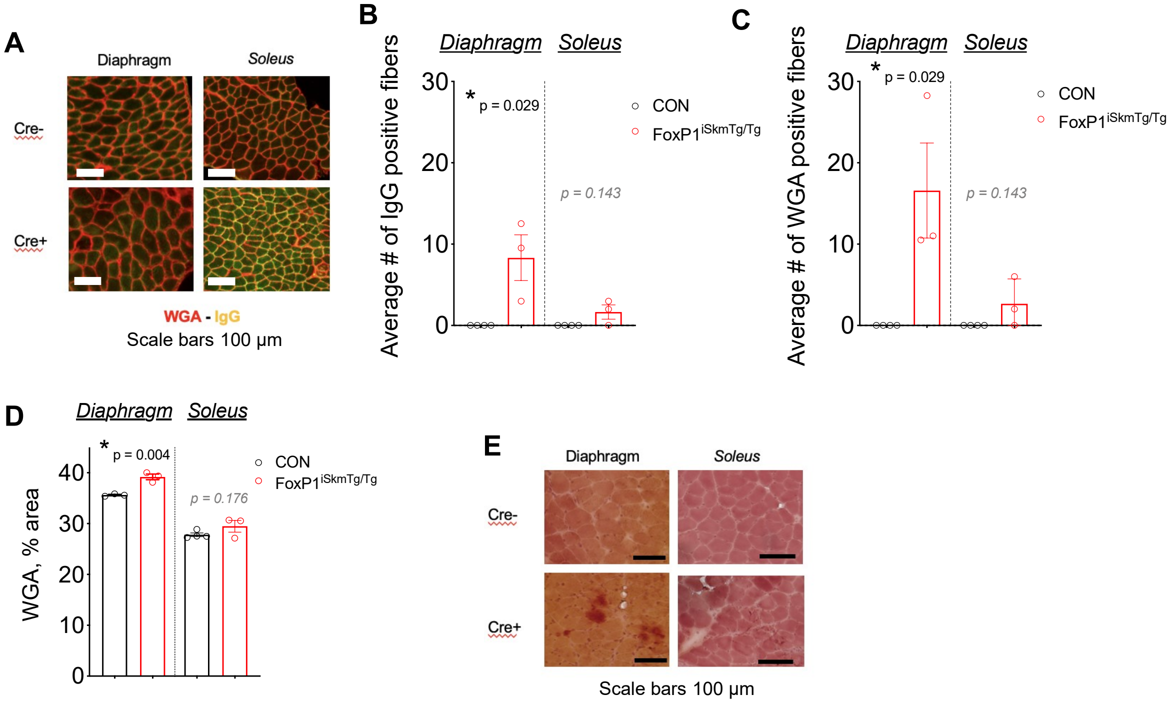

Figure S6. Skeletal muscle-specific FoxP1 over-expression also leads to fiber damage in *male* mice. (**A**) Representative images of diaphragm and *soleus* skeletal muscle cross sections from FoxP1$^{iSkmTg/Tg}$ mice and CRE- littermate controls (CON) stained with wheat germ agglutinin (WGA) and endogenous immonuglobulin G (IgG). (**B-D**) Quantification of IgG (**B**) and WGA (**C**) positive fibers as well as of the percent area showing WGA staining per 10X field of view (**D**). (**E**) Representative images of diaphragm and *soleus* skeletal muscle cross sections from FoxP1$^{iSkmTg/Tg}$ mice and CRE- littermate controls stained with Alizarin Red. Data are reported as mean ± SEM.

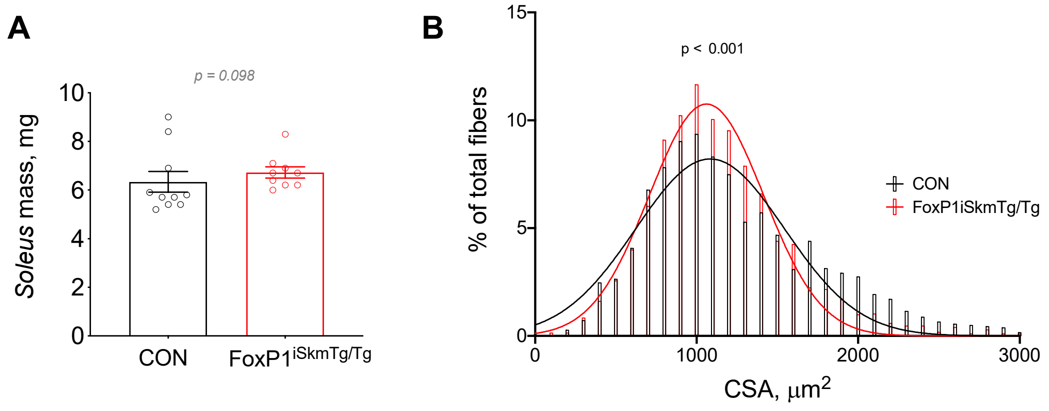

Figure S7. Female *soleus* (A) muscle mass and (B) fiber size distribution in response to skeletal muscle specific FoxP1 over-expression. Fiber cross-sectional areas (CSA) were binned, fit with a Gaussian least squares regression and significance was determined by calculating the extra sum-of-squares F test.

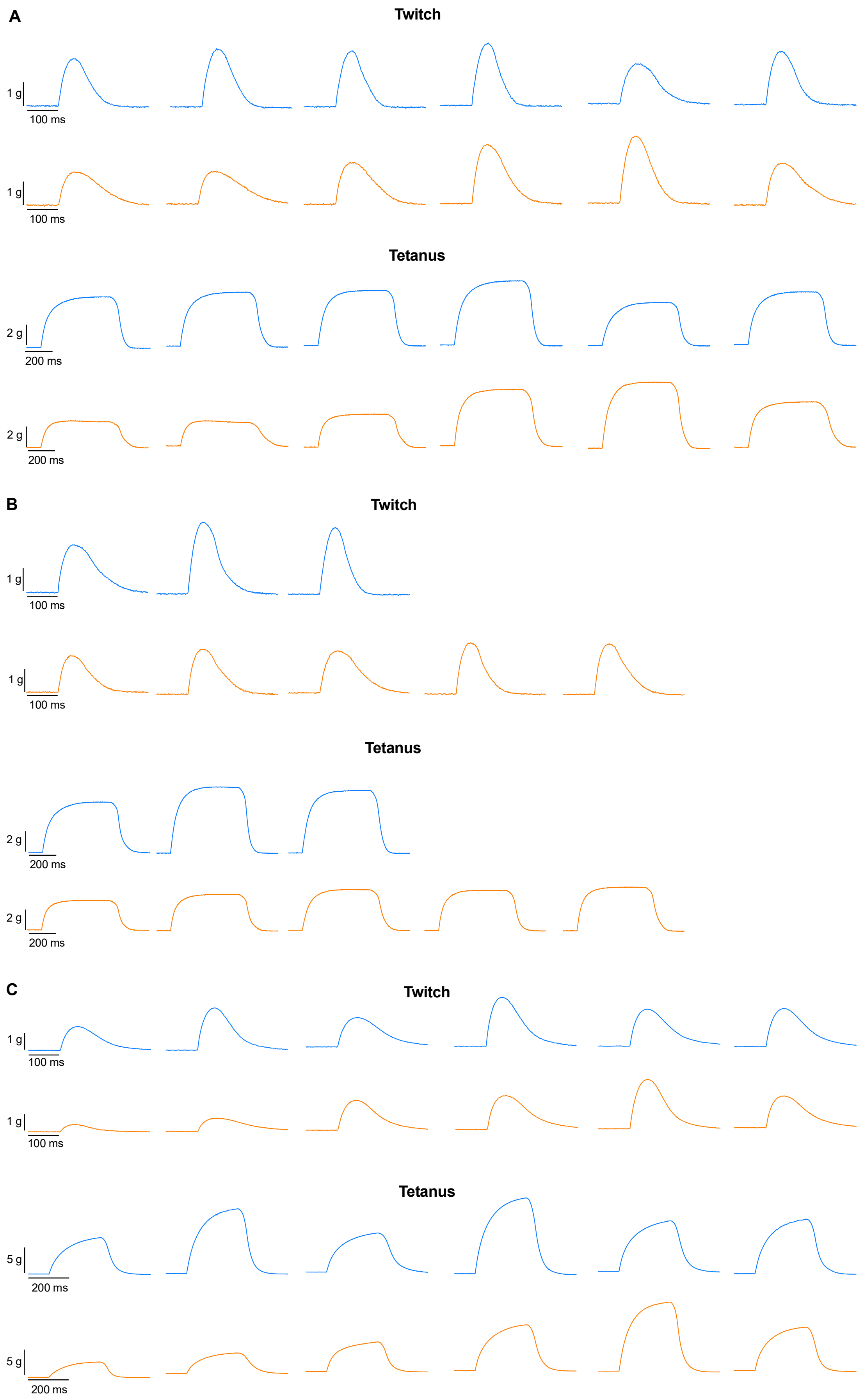

Figure S8. Original traces of twitch and tetanic forces recorded in (A) female and (B) male diaphragm strips as well as in (C) female *solei* of FoxP1$^{iSkmTg/Tg}$ (orange) and CRE- littermate (CON, blue) mice.

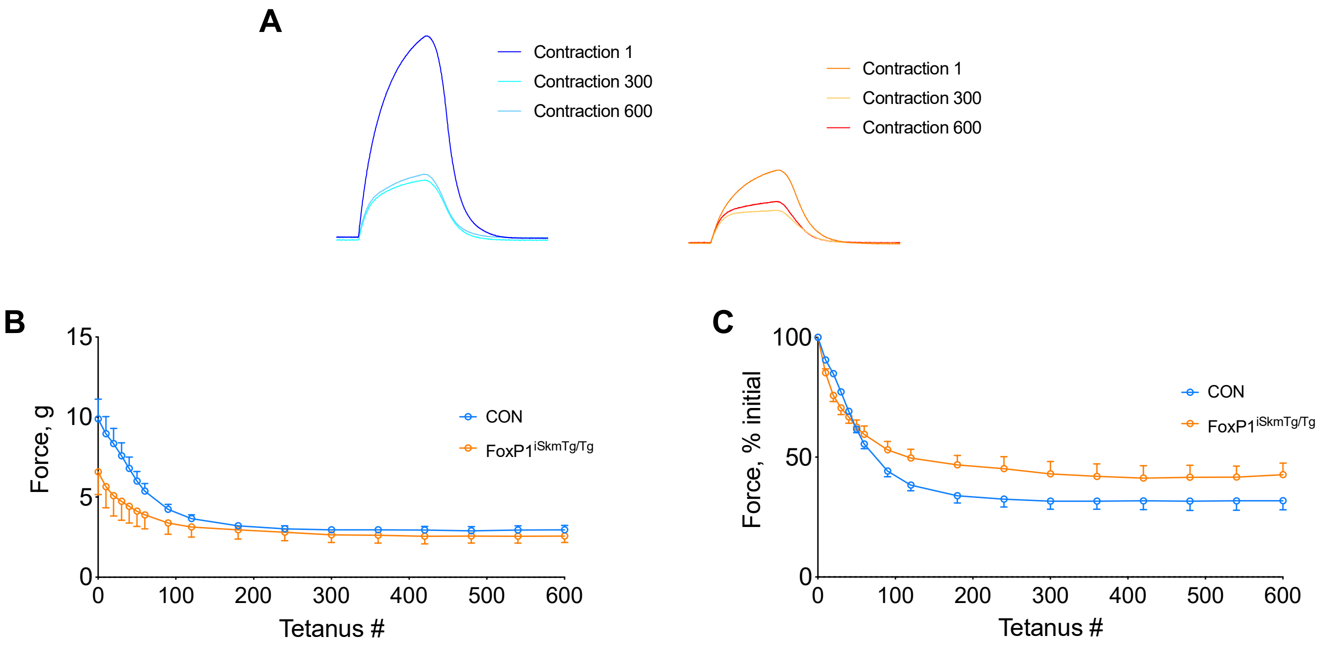

Figure S9. Skeletal muscle specific FoxP1 over-expression does not affect skeletal muscle fatigability. (**A**) Typical traces of force evoked by the 1st, 300th and 600th contraction and average of (**B**) absolute and (**C**) relative evoked forces in FoxP1$^{iSkmTg/Tg}$ (orange) and CRE- littermates (CON, blue). Data are reported as mean ± SEM.

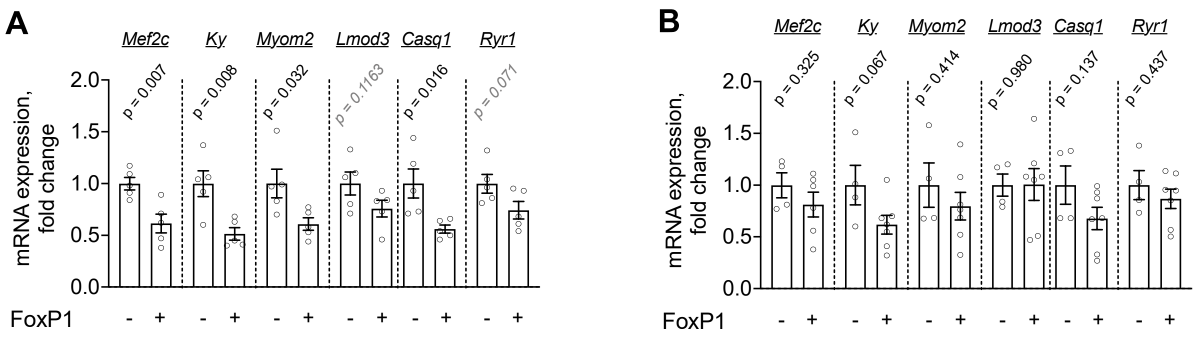

Figure S10. FoxP1 transcriptional repression is mediated, at least in part, by HDACs. (**A**-**B**) Mouse *tibialis anterior* muscles were transfected with a FoxP1 plasmid or empty vector. Subsequently, a subset of these mice received daily intraperitoneal (IP) injections of trichostatin A (TSA, a pan HDAC inhibitor) for four consecutive days (**B**). RT-qPCR shows that FoxP1-induced repression of several genes (**A**) is mediated by HDAC activity (**B**). Data are reported as mean ± SEM.
